# Priming a vascular-selective cytokine response permits CD8^+^ T-cell entry into tumors

**DOI:** 10.1038/s41467-023-37807-z

**Published:** 2023-04-14

**Authors:** Dae Joong Kim, Swetha Anandh, Jamie L. Null, Piotr Przanowski, Sanchita Bhatnagar, Pankaj Kumar, Sarah E. Shelton, Erin E. Grundy, Katherine B. Chiappinelli, Roger D. Kamm, David A. Barbie, Andrew C. Dudley

**Affiliations:** 1 Department of Microbiology, Immunology, and Cancer Biology, The University of Virginia, Charlottesville, VA 22908 USA; 2 Department of Biochemistry and Molecular Genetics, The University of Virginia, Charlottesville, VA 22908 USA; 3 Medical Microbiology and Immunology, The University of California Davis, School of Medicine, Davis, CA 95616 USA; 4 Department of Biological Engineering, Massachusetts Institute of Technology, Cambridge, MA 02139 USA; 5 Department of Medical Oncology, Dana-Farber Cancer Institute, Boston, MA 02215 USA; 6 Department of Microbiology, Immunology and Tropical Medicine, The George Washington University Cancer Center, The George Washington University School of Medicine and Health Sciences, Washington, DC USA; 7 Department of Mechanical Engineering, Massachusetts Institute of Technology, Cambridge, MA 02139 USA; 8 Belfer Center for Applied Cancer Science, Dana-Farber Cancer Institute, Boston, MA 02215 USA; 9 UVA Comprehensive Cancer Center, The University of Virginia, Charlottesville, VA USA

**Keywords:** Cancer microenvironment, Chemokines, Tumour immunology

## Abstract

Targeting DNA methyltransferase 1 (DNMT1) has immunomodulatory and anti-neoplastic activity, especially when paired with cancer immunotherapies. Here we explore the immunoregulatory functions of DNMT1 in the tumor vasculature of female mice. *Dnmt1* deletion in endothelial cells (ECs) impairs tumor growth while priming expression of cytokine-driven cell adhesion molecules and chemokines important for CD8^+^ T-cell trafficking across the vasculature; consequently, the efficacy of immune checkpoint blockade (ICB) is enhanced. We find that the proangiogenic factor FGF2 promotes ERK-mediated DNMT1 phosphorylation and nuclear translocation to repress transcription of the chemokines *Cxcl9/Cxcl10* in ECs. Targeting *Dnmt1* in ECs reduces proliferation but augments Th1 chemokine production and extravasation of CD8^+^ T-cells, suggesting DNMT1 programs immunologically anergic tumor vasculature. Our study is in good accord with preclinical observations that pharmacologically disrupting DNMT1 enhances the activity of ICB but suggests an epigenetic pathway presumed to be targeted in cancer cells is also operative in the tumor vasculature.

## Abstract (web summary)

It has been reported that inhibition of the epigenetic regulator DNA methyltransferase 1 (DNMT1) is associated with improved response to immune checkpoint blockade (ICB). Here the authors show that conditional deletion of Dnmt1 in endothelial cells is sufficient to promote T cell infiltration, reduce tumor growth and enhance ICB response in preclinical models.

## Introduction

Immune checkpoint blockade (ICB) has been transformative for the treatment of multiple cancer types; however, because not all patients respond to ICB, combinatorial therapeutic approaches are being explored. For example, epigenetic modifying drugs are known to improve ICB efficacy through diverse mechanisms^1–7^. These mechanisms include the unmasking of immunogenic endogenous retroviruses (ERVs) that are epigenetically silenced, stimulation of Th1 chemokine expression, and enhanced expression of MHC, thereby augmenting the presentation of neoantigens to antigen-presenting cells (APCs)^8,9^. The promising results reported in preclinical models using combinations of ICB and drugs that target the epigenome has spurred the initiation of new clinical trials for multiple cancer types. The success of these approaches depends, in part, on how well anti-tumor immune cells, including cytotoxic T-lymphocytes (CTLs), natural killer (NK) cells, and various antigen-presenting cells such as dendritic cells (DCs) can access the tumor microenvironment (TME). The TME is known to present barriers to effective ICB through diverse mechanisms. Due to its sentinel-like role and principal passageway for CTLs in the TME, the tumor endothelium is one such barrier.

Leukocytes such as CTLs enter tumors or lymph nodes predominately via rolling, adhesion, and migration across post-capillary venules or specialized blood vessels known as high endothelial venules^10^. Cross-talk between circulating leukocytes and the endothelium requires the expression of multiple homing receptor ligands (HRLs) (e.g., CXCL9 and CXCL10) that guide CTLs towards the TME, and cell adhesion molecules (e.g., VCAM1 and E-selectin) that mediate tethering to the vasculature followed by extravasation^11,12^. However, several recent studies suggest that tumor-associated endothelial cells (TECs) show suppression of CAMs and other factors that lead to impaired recruitment, retention, or survival of tumor-infiltrating lymphocytes (TILs)^13–20^. As a corollary, targeting tumors with low doses of anti-angiogenic or other therapies, or using genetically engineered mice to modify the expression of a target gene, can prime the vasculature to generate activated endothelium that is more receptive to cancer-killing immune cells^21–31^. Due to their ability to regulate the expression of multiple genes in tandem, including CAMs and chemokines, epigenetic therapies have the potential to reprogram the tumor vasculature, boost tumor immune surveillance, and enhance CTL entry and/or retention.

DNA methyltransferase 1 (DNMT1) is an enzyme that epigenetically controls gene expression by catalyzing the addition of methyl groups to CpG sites resulting in gene silencing. DNMT1 is typically downregulated in differentiated cells/tissues but is re-expressed in multiple cancers where it promotes proliferation and self-renewal; particularly in cancer-initiating cells^32,33^. High intratumoral DNMT1 levels also associate inversely with numbers of CD8^+^ CTLs, and it was recently shown that DNMT1 inhibition augments ICB through different mechanisms^4^. Collectively, these studies show important roles for DNMT1 during anti-tumor immunity, but it is unknown how methylation-specific cues in other cell types in the tumor microenvironment might impact tumor immune surveillance and responses to ICB.

In this work, to further define the precise mechanisms underlying epigenetic modulation of anti-tumor immunity in the tumor microenvironment, we generate a conditional deletion model to disable DNMT1 activity, specifically in the endothelium. By doing so, we uncover a role for DNMT1 in tumor blood vessels, including influences on tumor growth, microvessel patterning, and regulation of CAMs and chemokines that permit T-cell trafficking across the vasculature. We also identify a fibroblast growth factor-2 (FGF2) mediated mechanism for DNMT1 regulation, thus linking a potent endothelial cell (EC) mitogen with proliferative yet immunologically anergic tumor vasculature.

## Results

### Conditional deletion of Dnmt1 in ECs impairs tumor growth and reduces vascular density

**Fig. 1:**
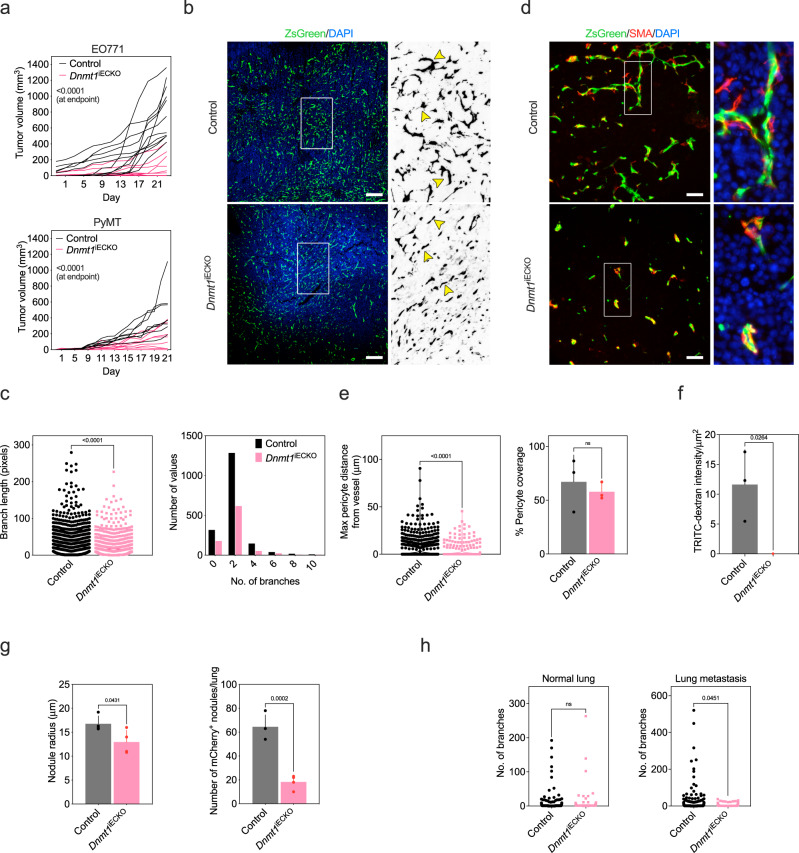
Conditional deletion of *Dnmt1* in ECs impairs tumor growth and reduces vascular density. **a** EO771 (top) or PyMT (bottom) mammary tumors orthotopically engrafted in control versus *Dnmt1*^iECKO^ mice. Tumor volumes were measured every other day with calipers (EO771; *n* = *11*, control and *n* = *9*, *Dnmt1*^iECKO^:PyMT; *n* = *8*, control and *n* = *8*, *Dnmt1*^iECKO^). For EO771, results were statistically significant beginning at day 15; for PyMT results were statistically significant beginning on day 16. Results were analyzed using ANOVA and Sidak’s multiple comparisons test. **b** ZsGreen^+^ tumor vasculature from the indicated mice. Boxed areas at right were zoomed 5× for detail and converted to an 8-bit binary image, and then inverted to reveal lateral branches/filopodia. Arrowheads point to either highly branched or straighter and narrower vessels in control versus *Dnmt1*^iECKO^ mice, respectively. Scale bars = 100 μm. **c** Quantification of vessel branch length presented as a dot plot or histogram to show the distribution of branch lengths and numbers of branches (*n* = *3* individual tumors and nine histological sections per group). Results were analyzed using Student’s *t* test. **d** Identification of pericytes in tumors using αSMA. Boxed areas were zoomed 5× and are shown at far right for detail. Scale bars = 100 μm. **e** Quantification of pericyte:EC distance (left) and total pericyte coverage (right) for control versus *Dnmt1*^iECKO^ mice (*n* = *3* individual tumors and nine histological sections per group). Results were analyzed using Student’s *t* test. **f** Extravascular TRITC-dextran quantified from cryosections using *n* = *3* mice per group. Results were analyzed using Student’s *t* test. **g** Tumor nodule radius (left) and total number of tumor nodules formed by mCherry^+^ EO771 cancer cells injected via the tail vein in the indicated mice (*n* = *4* mice per group). Results were analyzed using Student’s *t* test. **h** Number of vessel branches in areas of normal lung versus areas containing mCherry^+^ tumor nodules in the indicated mice (*n* = *3* individual tumors and nine histological sections per group). All Student’s *t* tests are unpaired and two-tailed. All data are presented as mean ± STD. Source data are provided as a Source Data file.

*Dnmt1* KO mice do not survive past mid-gestation due, in part, to a failure of hematopoiesis/yolk sac angiogenesis^34^. Thus, to determine the specific role of *Dnmt1* in the vasculature during tumor growth and immune surveillance, we generated a conditional deletion model to ablate *Dnmt1* in *Cdh5*^+^ ECs (*Dnmt1*^iECKO^ mice) (Supplementary Fig. 1a–d). Tumor growth was impaired when *Dnmt1* was disabled in ECs, but not in fibroblasts using *Postn-cre* mice, with orthotopically engrafted EO771 and PyMT mammary tumor cells (Fig. 1a, Supplementary Fig. 2a, b). Surprisingly, microvessel density was moderately reduced in these tumors, despite the robust inhibition of tumor growth; instead, when compared with tumors from control mice, the vasculature in tumors from *Dnmt1*^iECKO^ mice was narrower with fewer and shorter lateral branches (Fig. 1b, c, Supplementary Fig. 3). This was accompanied by a reduced EC-to-pericyte distance, an indicator of vessel normalization, although the total percentage of vessels with pericyte coverage was unchanged in tumors from *Dnmt1*^iECKO^ mice versus controls (Fig. 1d, e). To assess the functionality/perfusion of tumor blood vessels in the different genetic backgrounds, we injected TRITC-dextran via the tail vein prior to euthanasia. The results showed a characteristic extra-vascular leakage of TRITC-dextran in tumors from control mice that was diminished in *Dnmt1*^iECKO^ mice; again an indication of vessel normalization (Fig. 1f, Supplementary Fig. 4). In a model of experimental metastasis, reduced intratumoral vessel branching accompanied an ~20% reduction in tumor nodule size and 3.5-fold reduction in tumor nodules/lung in *Dnmt1*^iECKO^ mice versus controls (Fig. 1g, Supplementary Fig. 5a). Of note, vessel densities were no different in regions of normal lung tissues in *Dnmt1*^iECKO^ mice versus control mice suggesting that *Dnmt1* is not operative in quiescent, pre-existing vasculature, but may instead be important during the proliferation and migration of ECs as they incorporate into new vascular structures (Fig. 1h, Supplementary Fig. 5b). Taken together, genetic targeting of *Dnmt1* in ECs strikingly inhibits tumor growth despite subtle changes in vessel morphology and moderate reductions in vessel branch length; we therefore considered secondary mechanisms whereby loss of *Dnmt1* in ECs could impair tumor progression.

### Targeting Dnmt1 in EC cultures potentiates a cytokine response that augments vascular cell:T-cell interactions

DNA hypomethylating agents such as 5-Azacytidine (5-Aza) that inhibit DNMT1 were previously shown to potentiate the expression of Th1 chemokines that are important for CTL entry into tumors^4^. Since the tumor vasculature is a point-of-entry for anti-tumor immune cells, we have extended this observation by focusing on EC expression of cell adhesion molecules (CAMs) and chemokines after treating primary cultures of murine mammary gland ECs (MGECs) with 5-Aza in the presence or absence of the pro-inflammatory cytokines TNFα or IFNγ. By itself, 5-Aza resulted in a change in EC morphology that typifies “activated” ECs; moreover, increasing doses of 5-Aza strongly enhanced the expression *Vcam1*, *Esel*, *Cxcl9*, and *Cxcl10* when added with TNFα (Supplementary Fig. 6a, b). By comparison, expression of *Icam2*, *Vcam1*, and *Cxcl9* was enhanced when 5-Aza was added with IFNγ (Supplementary Fig. 6b). Because higher doses of 5-Aza appeared to be cytotoxic which diminished its potentiating effects on CAM/chemokine expression, we assayed the activity of GSK3484862 (a non-covalent inhibitor of DNMT1) in the presence of TNFα or IFNγ and found that it produced comparable results to 5-Aza (Supplementary Fig. 6b). These results suggest that optimal inhibition of DNMT1 in ECs primes an upregulation of cytokine-induced CAMs and chemokines that signal via divergent mechanisms.

**Fig. 2:**
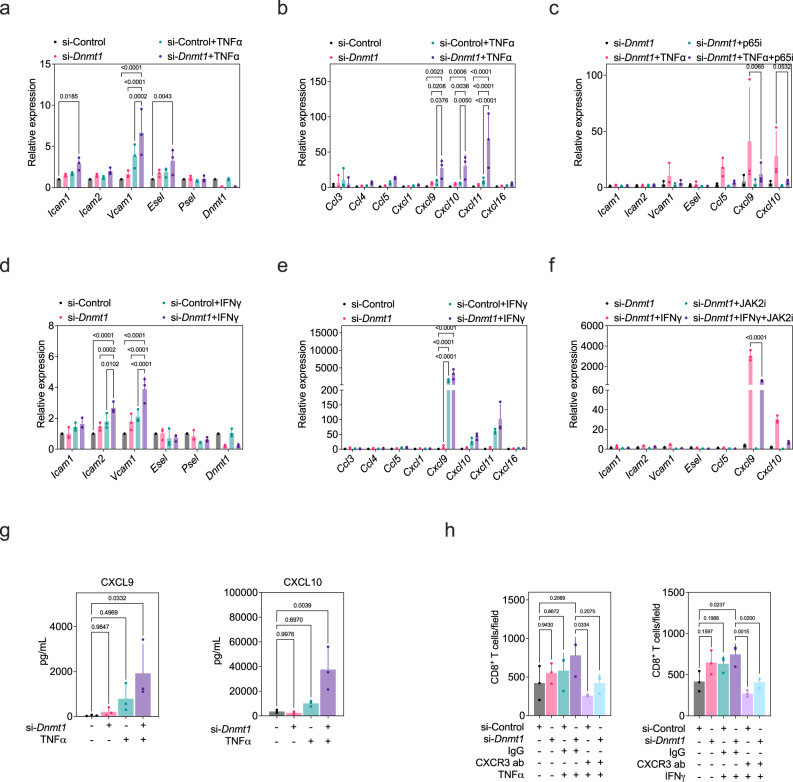
Targeting Dnmt1 in EC cultures potentiates a cytokine response that augments vascular cell:T-cell interactions. **a** MGECs were treated with scrambled control or *Dnmt1* siRNA prior to challenge with TNFα. Selected CAMs were analyzed by qPCR. **b** Same as in “**a**” except selected chemokines were analyzed by qPCR. **c** Same as in “**a**, **b**” except cells were pre-treated with a p65 inhibitor prior to TNFα challenge. **d** MGECs were treated with scrambled control or *Dnmt1* siRNA prior to challenge with IFNγ. Selected CAMs were analyzed by qPCR. **e** Same as in “**d**” except selected chemokines were analyzed by qPCR. **f** Same as in “**d**, **e**” except cells were pre-treated with a JAK2 inhibitor prior to IFNγ challenge. Each data point on the qPCR plots is the mean of *n* = *3* biological replicates run in triplicate. All qPCR data were analyzed using ANOVA followed by Tukey’s multiple comparisons test. **g** Luminex analysis of CXCL9/CXCL10 in concentrated MGEC conditioned media following scrambled control or *Dnmt1* siRNA ± TNFα challenge. Each data point is the mean of *n* = *3* biological replicates run in duplicate. Data were analyzed using ANOVA followed by Dunnett’s multiple comparisons test. **h** Adhesion assays using activated CD8^+^ T-cells incubated with MGECs under the indicated conditions. Each data point is the mean of *n* = *3* biological replicates run in triplicate. Data were analyzed using ANOVA followed by Tukey’s multiple comparisons test. All error bars are mean ± STD. Source data are provided as a Source Data file.

5-Aza can inhibit the activity of all three DNMT1 proteins. Although we found very low levels of *Dnmt3a*/*Dnmt3b* in ECs when compared to *Dnmt1*, we used a siRNA approach to specifically ablate *Dnmt1* expression in EC cultures (Supplementary Fig. 7a). Silencing *Dnmt1* did not impact the expression of *Dnmt3a/3b*, but approximated the effect of 5-Aza resulting in an ~2–4-fold increase in TNFα-stimulated *Icam1*, *Vcam1*, and *Esel* and ~25–100-fold increases in *Cxcl9/10/11* which are important chemo-attractants for guiding CXCR3^+^ T-cells towards the tumor microenvironment (Supplementary Fig. 7b, Fig. 2a, b). Notably, silencing HDAC2, which is known to physically associate with DNMT1 to epigenetically regulate transcription, blocked the potentiation of *Cxcl9/Cxcl10* expression associated with *Dnmt1* loss; these results suggest that HDAC2 positively regulates the expression of these chemokines once DNMT1 is no longer present (Supplementary Fig. 7c)^35^. Although the T-cell attracting chemokine *Ccl5* was also modestly enhanced when *Dnmt1* was silenced alongside TNFα addition, the effect of *Dnmt1* silencing was mostly selective for CXCR3-binding chemokines (*Cxcl9/10/11*) as an unbiased, high-throughput screen of the conditioned media from *Dnmt1*-silenced/TNFα-treated ECs showed abundant CXCL9/10/11 protein secretion when compared to other secreted factors (Supplementary Fig. 8a, b).

Since TNFα is well-known to upregulate the expression of CAMs/chemokines in ECs via NFκΒ, we asked whether NFκΒ inhibition would reverse the potentiation in CAM/chemokine expression that was primed by *Dnmt1* silencing. Blocking the NFκΒ subunit p65 partially dampened the augmented expression of *Vcam1*, *Ccl5*, *Cxcl9*, and *Cxcl10* in the context of *Dnmt1* silencing and TNFα stimulation (Fig. 2c). Using an identical strategy with IFNγ stimulation, we found that *Dnmt1* silencing, in this case, potentiated upregulation of *Icam2*, *Vcam1*, and *Cxcl9*; expression of *Cxcl10/11* were trending upward when *Dnmt1* was silenced alongside IFNγ stimulation but did not reach statistical significance possibly due to the variable magnitude of expression between multiple experiments (Fig. 2d, e). Because IFNγ is well-known to require JAK2 for signal transduction, we blocked JAK2 using the pharmacological inhibitor AG490. As before, JAK2 inhibition partially reduced the augmented expression of *Vcam1*, *Cxcl9*, and *Cxcl10* (Fig. 2f). Possibly owing to the short-term duration of these in vitro experiments and despite the now well-established role of DNA methylation during the suppression of IFN response genes driven by unmasking of endogenous transposable elements (TEs), we did not find a consistent upregulation of several candidate TEs in ECs challenged with TNFα or IFNγ ± *Dnmt1* silencing (Supplementary Fig. 9a, b)^8,36^. Taken together, these results suggest that *Dnmt1* silencing exerts its effects, in part, via hyper-activation of canonical signaling pathways downstream of TNFα and IFNγ.

*Dnmt1* silencing also increased the surface expression of TNFα-induced E-selectin and IFNγ-induced VCAM-1, but these increases were modest, likely because cytokine stimulation by itself results in large-magnitude increases in the expression of these factors (Supplementary Fig. 10a–d); by contrast, *Dnmt1* silencing resulted in several-fold increases in secreted CXCL9/CXCL10, that was well-above TNFα stimulation on its own, as measured by Luminex assays (Fig. 2g). Introduction of full-length murine *Dnmt1* suppressed both the TNFα- and IFNγ- driven expression of *Cxcl9* and *Cxcl10* and, as expected, TNFα or IFNγ reduced the enrichment of DNMT1 or total methylation (5mC) on *Cxcl9* and *Cxcl10* promoters using chromatin immunoprecipitation (ChIP) (Supplementary Fig. 11a–c). Notably, in human breast cancers, the expression of *CXCL9/10/11* shows strong positive associations with *CD8* expression (used as a surrogate for intratumoral T-cell content), which is consistent with a recent study (Supplementary Fig. 12a, b)^1^. In human breast cancer patients, higher numbers of CD8^+^ T cells or CXCR3^+^ T cells also associated with improved overall survival (Supplementary Fig. 12c, d). These data are in good accord with the suggestion that strategies to further enhance the crosstalk between vascular cells and CD8^+^ T-cells could improve anti-tumor immunity by overcoming an otherwise non-permissive tumor vasculature^37^. However, it should be noted that contradictory results are also reported with regard to CXCR3 expression and survival of human breast cancer patients likely due to the reported expression of CXCR3 on breast cancer cells which can promote CXCL9/10-mediated motility^38,39^. Thus, we assayed the direct functional impact of *Dnmt1* silencing in MGECs with respect to CD8^+^ T-cell adhesion. We found that *Dnmt1* silencing augmented TNFα- and IFNγ- stimulated adhesion of CellTracker^TM^ green-labeled, CD3/CD28-activated splenic CD8^+^ T-cells to MGEC monolayers relative to cytokine stimulation alone (Fig. 2h). Inclusion of CXCR3 neutralizing antibodies reduced CD8^+^ T-cell adhesion to MGEC monolayers in the context of cytokine stimulation ± *Dnmt1* silencing. Notably, conditioned media collected from MGEC treated with *Dnmt1* siRNA and TNFα was sufficient to promote T-cell adhesion to secondary MGEC monolayers suggesting a paracrine-mediated mechanism (Supplementary Fig. 13a, b). These results are consistent with a reported CXCL9/CXCL10/CXCR3 axis in driving T cell:vascular cell adhesion in tumors suggesting a functional link between potentiation of EC-derived CXCL9/CXCL10 expression by *Dnmt1* silencing and enhanced CD8^+^ T-cell trafficking^40^.

### Conditional deletion of Dnmt1 in ECs reprograms the tumor vasculature resulting in increased numbers of intra-tumoral and perivascular CD8^+^ T-cells and increased GzB^+^ cells

**Fig. 3:**
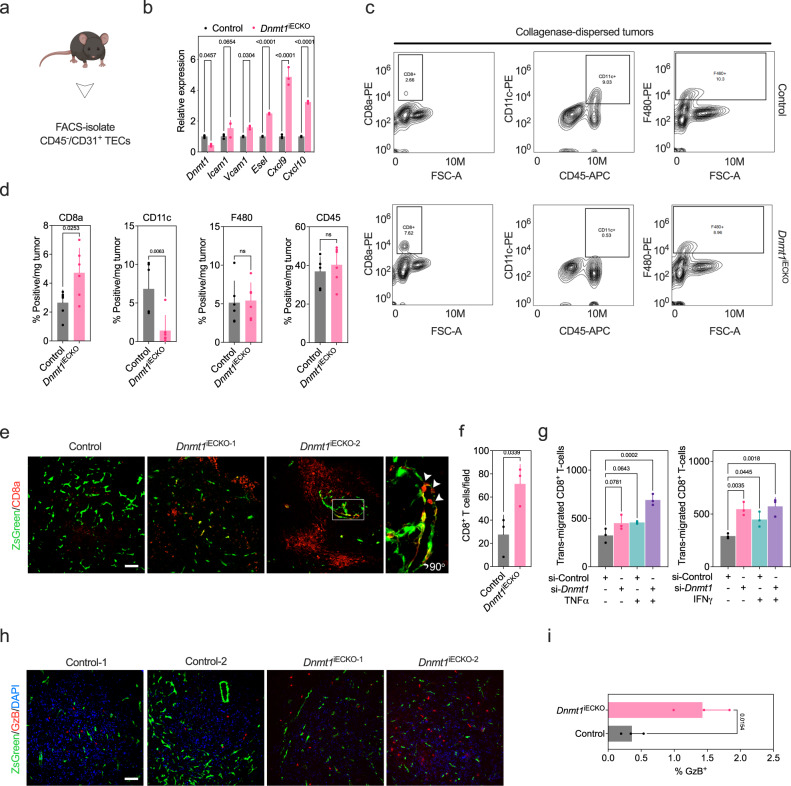
Conditional deletion of *Dnmt1* in ECs reprograms the tumor vasculature resulting in increased numbers of intra-tumoral and perivascular CD8^+^ T-cells and increased GzB^+^ cells. **a** Orthotopically injected EO771 mammary tumors were dissociated with an enzyme cocktail. CD45^−^/CD31^+^ TECs were isolated and analyzed using FACS (*n* = *3* pooled tumors from the indicated group of mice). **b** qPCR analysis of selected target genes in TECs assayed in triplicate. Data were analyzed using ANOVA followed by Sidak’s multiple comparisons test. **c** Same as in “**a**” except the indicated antibody combinations were used to identify immune cell subsets (representative contour plots are shown) in collagenase-dispersed tumors. **d** Quantification of FACS analysis shown in “**c**” (*n* = *6* mice/group) and each data point is an individual mouse. Data were analyzed using Student’s *t* test. **e** Cryosections of EO771 mammary tumors from control versus *Dnmt1*^*iECKO*^ mice stained with anti-CD8 antibodies (red fluorescence). Vessels are marked with ZsGreen. The images show representative fields of view of “small” (*Dnmt1*^iECKO^-1) versus “large” (*Dnmt1*^iECKO^-2) CD8^+^ T-cell clusters and peri- or intra-vascular T-cells (boxed area zoomed 5× shown at far right). Scale bar = 50 μm. **f** Quantification of CD8^+^ T-cells/field of view (*n* = *3* tumors from each group). Results were analyzed using Student’s *t* test. **g** Trans-endothelial migration assay using MGECs co-cultured with CD8^+^ T-cells under the indicated conditions. Each data point is the mean of *n* = *3* biological replicates run in triplicate. Results were analyzed using ANOVA followed by Dunnett’s multiple comparisons test. **h** Cryosections of EO771 mammary tumors from control versus *Dnmt1*^*iECKO*^ mice stained with GzB antibodies (red fluorescence). Vessels are marked with ZsGreen and nuclei (blue) are marked with DAPI. The images show two representative examples of tumors from control versus *Dnmt1*^iECKO^ mice. Scale bar = 50 μm. **i** Quantification of percent GzB^+^ cells from *n* = *3* tumors in each group using *n* = *4* cryosections from each mouse. Results were analyzed using Student’s *t* test. All Student’s t-tests are unpaired and two-tailed. All error bars are mean ± STD. Source data are provided as a Source Data file.

Next, we used FACS to isolate tumor-associated endothelial cells (TECs) by gating on CD45^-^/CD31^+^ cells from collagenase-dispersed, orthotopically-engrafted EO771 mammary tumors. We confirmed upregulation of *Vcam1*, *Esel*, *Cxcl9*, and *Cxcl10* using RT-qPCR in TECs from *Dnmt1*^iECKO^ mice versus controls (Fig. 3a, b). VCAM-1 staining using fresh cryosections from control versus *Dnmt1*^iECKO^ tumors confirmed an increase in VCAM-1 that was detectable at the luminal surface (Supplementary Fig. 14a). Interestingly, we also found an increase in the number of vessels staining positive with the MECA-79 antibody that recognizes sulfated carbohydrate epitopes present on high endothelial venules which are specialized vessels important for T-cell entry into tumors (Supplementary Fig. 14b, c)^10^. Thus, we assessed whether *Dnmt1* silencing could augment the expression of HEV marker genes stimulated by Light and lymphotoxin, which are known inducers of HEV neogenesis^41^. The results showed that *Dnmt1* silencing in MGEC cultures was sufficient to enhance the expression of *Il33*, *Ccl21*, and *Ccl19*. Other genes such as *Madcam*, *Ackr1*, and *Chst4* were significantly augmented only relative to controls suggesting a direct role for DNMT1 in the regulation of selective genes that identify HEV ECs (Supplementary Fig. 14d).

Using FACS to identify different immune cell populations, we found an ~2–3-fold increase in CD8^+^ T-cells in tumors from *Dnmt1*^iECKO^ mice versus control mice (Fig. 3c, d). Surprisingly, CD11c^+^ dendritic cells were reduced by almost ~5-fold in *Dnmt1*^iECKO^ mice, whereas total numbers of F480^+^ macrophages or CD45^+^ hematopoietic cells were no different in the two groups of mice, suggesting a selective impact on specific immune cell populations. Since the numbers of CD11c^+^ cells were reduced in tumors, we hypothesized there could be an increase in CD11c^+^ cells trafficking to the draining lymph nodes where antigen presentation occurs. Indeed, immunohistochemistry of these lymph nodes revealed an ~3-fold increase in CD11c^+^ cells in *Dnmt1*^iECKO^ mice versus controls (Supplementary Fig. 15). Fresh cryosections from mammary tumors also confirmed a higher abundance of CD8^+^ T-cells in *Dnmt1*^iECKO^ mice, which appeared as large clusters throughout the TME, or were conspicuously tethered to the vascular wall which is suggestive of enhanced diapedesis (Fig. 3e, f). To test this possibility, we carried out diapedesis assays using a trans-well culture system consisting of MGEC monolayers co-cultured with CellTracker^TM^ green-labeled, CD3/CD28-activated splenic CD8^+^ T-cells, as above. The results show that *Dnmt1* silencing augmented TNFα- or IFNγ-stimulated trans-endothelial migration by ~2-fold when compared to controls (Fig. 3g). Finally, we examined numbers of granzyme B-expressing (GzB^+^) effectors as an indicator of recent antigen stimulation/activation and cytolytic function. Similar to total CD8^+^ T-cells, we found an ~4-fold increase in GzB^+^ cells in tumors implanted in *Dnmt1*^iECKO^ mice relative to control mice (Fig. 3h, i). Taken together, *Dnmt1* silencing in ECs produces whole-scale changes in the tumor immune microenvironment, including an unexpected reduction in CD11c^+^ dendritic cells, increases in CD8^+^ T-cells, including those associated with and extravasating the vasculature, and significant increases in GzB^+^ cytolytic cells.

### CD8-blocking antibodies rescue tumor growth, whereas ICB efficacy is enhanced in Dnmt1^iECKO^ mice

**Fig. 4:**
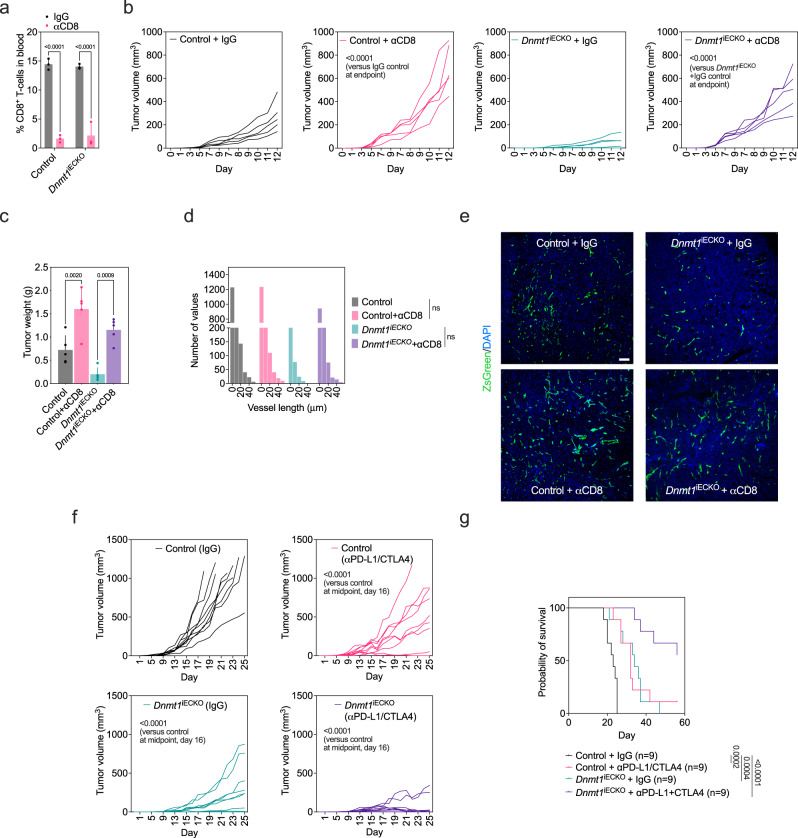
CD8-blocking antibodies rescue tumor growth, whereas ICB efficacy is enhanced in Dnmt1^iECKO^ mice. **a** Validation of depletion of CD8^+^ cells in blood drawn from the indicated mice (*n* = *3*). Data were analyzed by ANOVA followed by Sidak’s multiple comparisons test. **b** Tumor growth analysis in control versus *Dnmt1*^*iECKO*^ mice treated with IgG control or CD8-blocking antibodies (*n* = *5*). Results were analyzed using ANOVA followed by Tukey’s multiple comparisons test. **c** Tumor weights at the end of the study. Results were analyzed using ANOVA followed by Tukey’s multiple comparisons test (*n* = *5*). **d** Histogram quantification of vessel branch lengths under the indicated treatments (*n* = *3* individual tumors and nine histological sections per group). Results were analyzed using ANOVA followed by Tukey’s multiple comparisons test. **e** Representative images of vascular structures following the indicated treatments (ZsGreen marks tumor vasculature and DAPI marks nuclei; blue). Scale bar = 50 μm. **f** Tumor growth in control versus *Dnmt1*^*iECKO*^ mice treated with IgG control or αPD-L1 + CTLA4 combinations (control, *n* = *9*; *Dnmt1*^iECKO^ + IgG, *n* = *9*; control + αPD-L1/CTLA4, *n* = *9*; *Dnmt1*^iECKO^ + αPD-L1/CTLA4, *n* = *9*). Data were analyzed using ANOVA followed by Tukey’s multiple comparisons test. **g** Kaplan–Meier analysis for the indicated treatments (Log-rank Mantel-Cox test *p* values are indicated on the graph). All error bars are mean ± STD. Source data are provided as a Source Data file.

To determine the specific contribution of CD8^+^ T-cells towards diminished tumor growth in *Dnmt1*^iECKO^ mice, we carried out an antibody depletion experiment using CD8-blocking antibodies. After four consecutive rounds of CD8-blocking antibody treatment, CD8^+^ T-cells were ablated in the circulation (Fig. 4a). We found that CD8-blocking antibodies promoted rapid acceleration of tumor growth in control mice resulting in some tumors reaching their maximum allowed dimensions (1000 m^3^) within 12 days (Fig. 4b). As expected, tumor growth was impaired in *Dnmt1*^iECKO^ mice versus controls; moreover, CD8-blocking antibodies almost completely rescued tumor growth in *Dnmt1*^iECKO^ mice, indicated by a restoration in tumor volume and tumor weight at the end of the experiment (Fig. 4b, c). The restorative effect of CD8-blocking antibodies in *Dnmt1*^iECKO^ mice was not associated with a statistically significant recovery of microvessel branch length, although there was a trending increase in vessel branch length distribution in *Dnmt1*^iECKO^ mice treated with CD8-blocking antibodies compared to *Dnmt1*^iECKO^ mice alone (Fig. 4d, e). Because the effectiveness of immune checkpoint blockade is dependent, in part, on the accessibility of immune cells to the TME, we asked whether ICB efficacy is enhanced when *Dnmt1* is genetically silenced in the vasculature. In contrast to CD8-blocking antibodies, ICB prolonged the time until tumors reached maximum dimensions by almost two weeks, resulting in tumors that were approximately half the size in *Dnmt1*^iECKO^ mice relative to their respective counterpart controls (Fig. 4f). This translated into a significant increase in overall survival in *Dnmt1*^iECKO^ mice treated with ICB, relative to IgG controls and all other groups (Fig. 4g). These data suggest that targeting a single gene in the endothelium can impart secondary, large-scale changes to the TME including significant alterations in the composition of anti-tumor immune cells and improved anti-tumor immunity.

### ScRNA-sequencing reveals proliferative TECs that are diminished in IFN and TNF response genes

**Fig. 5:**
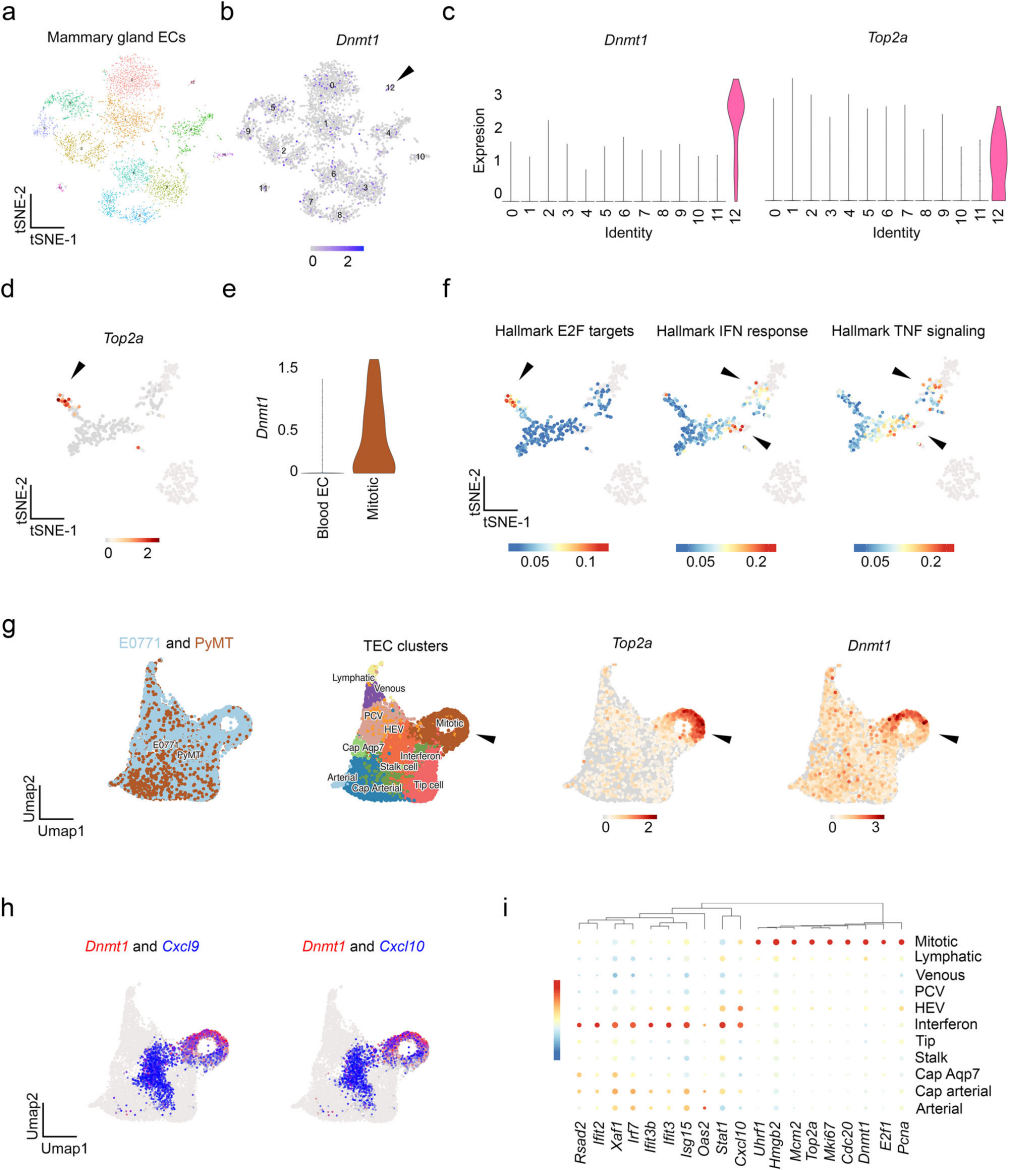
ScRNA-sequencing reveals proliferative TECs that are diminished in IFN and TNF response genes. **a** tSNE plot of normal mammary gland ECs isolated from pooled (*n* = *20*) mammary glands. **b** tSNE plot showing *Dnmt1* expression amongst the different clusters. The arrowhead points to cluster 12. **c** Violin plots showing enrichment of *Dnmt1* and *Top2a* in cluster 12. **d** tSNE plot showing *Top2a* expression in PyMT TECs. **e** *Dnmt1* expression in blood versus mitotic TECs from the PyMT model. **f** tSNEs depicting gene set enrichment scores for E2F targets (found in the mitotic cluster) and IFN or TNF response (found mainly in the non-mitotic clusters). **g** Clustering for E0771 and PyMT TECs including annotated clusters and *Top2a* or *Dnmt1* expression. The arrowhead points to the mitotic cluster. **h** Co-expression of *Dnmt1* and *Cxcl9* or *Cxcl10* in mitotic versus the IFN-like cluster. **i** Bubble plots for candidate IFN response genes and mitotic genes depicted by vessel subtype.

Tumor vasculature, like wounded vasculature, is known to display intra- and inter-vascular heterogeneity^42^. To further explore TEC heterogeneity as relates to *Dnmt1* expression and anti-tumor immune responses, we carried out scRNA-seq on FACS-isolated CD45^-^/CD31^+^ ECs from collagenase-dispersed, normal mammary glands. After filtering fractions of *Cd45*^*+*^ (*Ptprc*), *Myh11*^*+*^ (smooth muscle), *Epcam*^+^ (epithelial), and *Cspg4*^*+*^ (pericytes) cells, a tSNE plot shows the distribution of normal mammary gland ECs (NECs) (Supplementary Fig. 16a, b, Fig. 5a). We next scanned for *Dnmt1* expression and found that it was expressed at low levels throughout individual ECs in each cluster, but was also enriched in a single cluster (here called cluster 12) (Fig. 5b, c). Unbiased clustering of these ECs revealed that cluster 12 was also enriched in several markers of proliferation, including *Top2a*, indicating that cluster 12 is a mitotic cluster (Supplementary Fig. 16c, Fig. 5c). Using CD45^-^/CD31^+^ TECs from E0771 mammary tumors, we could also identify a mitotic cluster, marked by *Top2a*, that was enriched in *Dnmt1* relative to the other clusters; notably, this cluster was distinct from a second cluster that resembles a recently described IFN response EC cluster represented by genes such as *Irf8*, *Ifit2*, *Isg15*, and *Cxcl10* (Supplementary Fig. 17a–d)^41^.

Next, using publicly available datasets, we examined SmartSeq2 data from TECs using the PyMT mammary tumor model^41^. These data contain blood TECs in addition to lymphatic and HEV-like clusters. After filtering on blood TECs only, we identified a mitotic cluster and confirmed that *Top2a* was enriched; furthermore, *Dnmt1* was expressed in this mitotic cluster (Fig. 5d, e). *Dnmt1* is a known E2F target gene upregulated in mitotic cells, and we could confirm Hallmark E2F targets in the mitotic cluster that was distinct from cells enriched in the Hallmark IFN response or TNF signaling pathway (Fig. 5f). These data suggest that mitotic TECs, enriched in *Dnmt1*, may suppress genes important for IFN response or TNF signaling.

For a more comprehensive portrait of gene expression in TECs using the E0771 and PyMT models, we examined a second, well-annotated scRNAseq dataset (Fig. 5g)^41^. Again, we found *Top2a* and *Dnmt1* to be enriched in the mitotic cluster. Notably, whereas only 0.01% of normal mammary gland ECs expressed *Top2a*, as shown above, this number climbed to ~15% in mammary TECs as an indicator of enhanced mitosis overall. We then examined the co-expression of the IFN-response genes *Cxcl9* and *Cxcl10* versus *Dnmt1*. As expected, *Cxcl9*/*Cxcl10* were mostly enriched in the IFN cluster; we found that 25% of ECs co-expressed *Cxcl9*/*Dnmt1* and 17% co-expressed *Cxcl10*/*Dnmt1* (Fig. 5h). These percentages were lower when we examined their co-expression using the Smartseq2 data for PyMT TECs: only 5.8% of the cells co-expressed *Cxcl9*/*Dnmt1* and 1.7% co-expressed *Cxcl10*/*Dnmt1*. We finally plotted the IFN response and mitotic marker genes based on vessel subtype using E0771 and PyMT TECs and found them to be distinct (Fig. 5i). Taken together, *Dnmt1* is enriched in a small mitotic pool of ECs, and this cluster is distinct from clusters that express IFN-response genes important for regulating vascular/immune cell cross-talk. These results are consistent with a suppression of immunoregulatory pathways in proliferating cells in general, which is well-aligned with recent work describing enhanced efficacy of ICB in solid tumors when combined with cell cycle inhibitors^5,43^. Moreover, our data suggest that silencing *Dnmt1* may switch ECs from a mitotic to an IFN-responsive subtype.

### Targeting DNMT1 augments the adhesion of CXCR3-expressing T-cells to human 3D vascular networks under flow

**Fig. 6:**
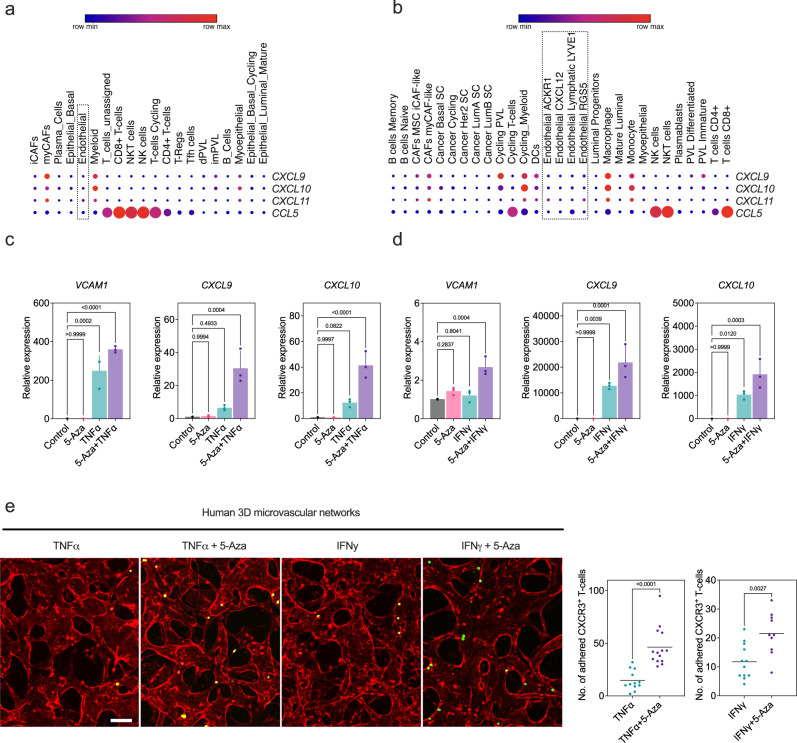
Targeting DNMT1 augments the adhesion of CXCR3-expressing T-cells to human 3D vascular networks under flow. **a** Examination of the indicated chemokine by cell type using human scRNAseq data from breast cancer. **b** Examination of the indicated chemokine by cell type using human scRNAseq data from breast cancer. Data from a-b were previously generated^44,45^. **c** qPCR analysis of the indicated target gene in human ECs (HUVEC) primed with 5-Aza prior to TNFα challenge (*n* = *3* biological replicates assayed in triplicate). Results were analyzed using ANOVA followed by Dunnett’s multiple comparisons test. **d** Same is in “**c**” except treatment with IFNγ. **e** Generation of perfusable 3D networks with human ECs under the indicated conditions. Thirty thousand CXCR3 over-expressing T-cells were labeled with CellTracker^TM^ green and were flowed through the 3D networks, washed, and imaged for analysis. Scale bar = 100 μm. At far right is quantification of adherent T-cells under the indicated conditions. Each dot represents an individual microfluidic device. Results were analyzed using an unpaired two-tailed Student’s *t* test and *p* values are shown on the graph. All error bars are mean ± STD. Source data are provided as a Source Data file.

To gain further insight into human TEC heterogeneity with regard to proliferation and IFN response genes, we accessed scRNA-seq data from two recent studies that comprehensively resolved the single-cell landscape of human breast cancers^44,45^. Our goal was to identify *TOP2A*- or *DNMT1*-enriched TECs that resembled our findings in the mouse mammary tumor models. While another recent study also found that *Top2a*^*hi*^ TECs were generally clustered as distinct from an IFN response cluster in murine lung, we were unable to identify a murine-equivalent association in these human studies^46^. However, what was apparent from these data is that human breast TECs express very low levels of Th1 chemokines in general, which was also found when we probed human lung TECs using publicly available datasets (Fig. 6a, b)^46^. These data point to key differences between murine tumor models versus human cancers and may relate to an accelerated rate of EC proliferation/angiogenesis that occurs using engrafted tumors in mice. Nevertheless, these data presented an opportunity to explore whether human ECs could also be primed to express abundant Th1 chemokines by targeting DNMT1. Indeed, we found that human umbilical vein endothelial cells (HUVECs) expressed low basal levels of Th1 chemokines, but their expression, along with *VCAM1*, could be potentiated by priming with 5-Aza prior to TNFα or IFNγ challenge (Fig. 6c, d).

To explore how inhibiting DNMT﻿1 in human ECs impacts functional interactions with immune cells, particularly CD8^+^ T cells, we generated 3D vascular networks with HUVECs using previously-established methodologies^47^. This in vitro system uses perfused, self-organizing networks of ECs and perivascular mural cells and flow rates that approximate human circulation. After networks were established, we primed with 5-Aza, followed by additional treatment with either TNFα or IFNγ. After infusing Jurkat cells over-expressing CXCR3 (CXCL9/10 receptor) through these networks, the results showed that 5-Aza addition enhanced T-cell adhesion to ECs ~2-fold higher when compared to cytokine treatment alone (Fig. 6e). These results are consistent with the concept that 5-Aza priming can potentiate T-cell adherence to human ECs, even under sheer stress/flow conditions.

### The potent EC mitogen FGF2 opposes DNMT1i-mediated potentiation of CAMs and T-cell- attracting chemokines

**Fig. 7:**
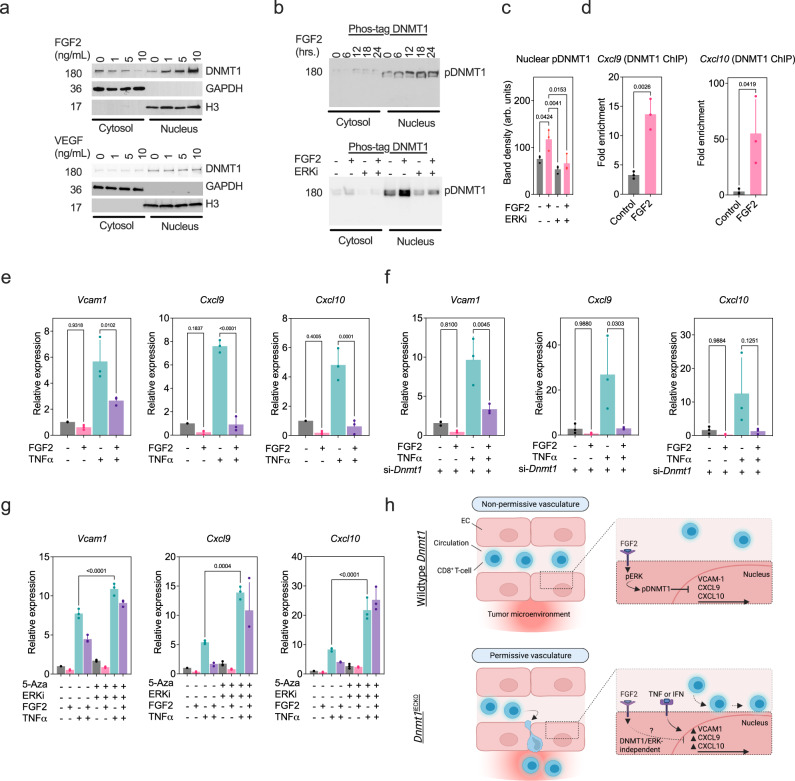
The potent EC mitogen FGF2 opposes *Dnmt1i*-mediated potentiation of CAMs and T-cell- attracting chemokines. **a** MGECs treated with the indicated concentration of FGF2 or VEGF were harvested, split into cytosolic or nuclear fractions, and then analyzed for the indicated proteins using SDS-PAGE followed by immunoblotting (representative of *n* = 3 independent blots). **b** MGECs treated with the indicated concentration of FGF2 were harvested, split into cytosolic or nuclear fractions, and then analyzed using Phos-tag gel electrophoresis (representative of *n* = 3 independent blots). **c** Quantification of nuclear pDNMT1 signal using *n* = *3* independent gels. Results were analyzed using ANOVA followed by Tukey’s multiple comparisons test. **d** ChIP assays in control versus FGF2-stimulated MGECs. ChIPs were performed using cross-linked whole-cell extracts incubated with DNMT1 antibodies. Captured DNMT1/DNA complexes were analyzed by qPCR to assess DNMT1 enrichment on *Cxcl9*/*Cxcl10* promoters in control versus FGF2-treated MGECs (*n* = *3* biological replicates). Results were analyzed using an unpaired two-tailed Student’s *t* test. **e** qPCR analysis for the indicated gene in MGECs treated as indicated; each data point is the mean of *n* = *3* biological replicates run in triplicate. **f** qPCR analysis for the indicated gene in MGECs treated as indicated; each data point is the mean of *n* = *3* biological replicate run in triplicate. **g** qPCR analysis for the indicated gene in MGECs treated as indicated; each data point is the mean of *n* = *3* biological replicate run in triplicate. **h** Schematic showing the role of an FGF2/DNMT1 axis in promoting a non-permissive tumor vasculature through suppression of CAMs and chemokines. Targeting DNMT1 results in a potentiation of TNF or IFN response genes that are important for anti-tumor immunity. ANOVA followed by Tukey’s multiple comparisons test was used to analyze results in panels **e**–**g**. All error bars are mean ± STD. Source data are provided as a Source Data file.

Because angiogenesis has been associated with immunosuppression, we hypothesized that EC mitogens such as FGF2 or VEGF might be immunosuppressive specifically through their ability to regulate the expression of *Dnmt1*. As expected, FGF2 stimulated the in vitro proliferation and angiogenic sprouting of ECs, but these effects were mitigated by *Dnmt1* silencing (Supplementary Fig. 18a, b). Furthermore, FGF2 stimulation reduced the numbers of CD8^+^ T-cells adhered to TNFα-stimulated MGECs (Supplementary Fig. 18c). We also found that FGF2 stimulation, but not VEGFA, dose-dependently increased DNMT1 translocation to the nucleus while depleting its expression in the cytoplasm (Fig. 7a, Supplementary Fig. 18d). DNMT1 is known to be enriched in proliferating cells in response to mitogens and DNMT1 deletion was shown to result in an upregulation of several inhibitory cyclin-dependent kinases in fibroblasts^48^; similarly, *Dnmt1* siRNAs in ECs upregulated *Cdkn1a* and *Cdkn1c* which could explain, in part, the defect in EC proliferation when *Dnmt1* is genetically targeted in the vasculature (Supplementary Fig. 18e).

Several serine/threonine residues within DNMT1’s regulatory domains were shown to be phosphorylated by protein kinase C (PKC) or extracellular signal-regulated kinases (ERKs), leading to protein stability^49,50^. ERK1/2 is well-known to be activated by FGF2 in ECs, and we observed increases in phosphorylated nuclear DNMT1 following FGF2 stimulation that was inhibited by blocking ERK1/2 with a highly selective inhibitor (ERKi) (Fig. 7b, c, Supplementary Fig. 18f). Given these data, we hypothesized that FGF2 is immunosuppressive in ECs, in part, via direct regulation of DNMT1 expression/nuclear translocation. We found that FGF2 stimulation resulted in 3–15-fold enrichment for DNMT1 on the promoters of *Cxcl9* and *Cxcl10* (Fig. 7d). Furthermore, pre-treating ECs with FGF2, prior to TNFα challenge, resulted in several-fold reductions in expression of *Vcam1*, *Cxcl9*, and *Cxcl10*; in contrast, VEGF stimulation resulted in modest suppression of these same factors (Fig. 7e, Supplementary Fig. 18g).

Finally, we tested the relative contribution of DNMT1 in mediating the immune suppressive effects of FGF2 in the presence of TNFα. Surprisingly, silencing *Dnmt1* prior to FGF2/TNFα challenge could not mitigate the suppressive effect of FGF2 on its own (Fig. 7f). These results suggest that DNMT1 may impart stable methylation marks that are removed gradually from the regulatory sequences of the candidate genes analyzed and/or FGF2 can promote EC anergy via activation of alternative but complimentary repressive pathways^51^. Reasoning that siRNAs only partially reduce *Dnmt1* levels or that low levels of *Dnmt3a*/*3b* expression might compensate for selectively-targeted *Dnmt1*, we used 5-Aza to inhibit DNMT1 activity alongside ERKi. Combinatorial blockade of both DNMT1 and ERK1/2 again partially antagonized FGF2’s ability to suppress TNFα-induced *Vcam1* and *Cxcl9* but completely antagonized *Cxcl10* suppression; moreover, blocking both DNMT1 and ERK1/2 resulted in a significant increase in *Vcam1*, *Cxcl9*, and *Cxcl10* mRNAs (Fig. 7g). Taken together, these data suggest that FGF2, through both DNMT1/ERK-dependent and -independent pathways, potently antagonizes the expression of EC CAMs and chemokines that are important for CTL homing and entry into tumors or tumor-draining lymph nodes (Fig. 7h).

## Discussion

In tumors, clonally-derived subpopulations of ECs are suggested to suppress Th1 chemokines and CAMs that are important for immune surveillance^13,14,18^. Recent studies also suggest that in sites of injury or in tumors, ECs are derived from highly proliferative and hierarchical progenitors that undergo transient shifts in EC fate, including a return to a more primitive state that permits re-entry into the cell cycle^52–56^. Our studies in mice reveal an inverse association between proliferation and activation of IFN response genes; thus, it is possible that ECs in tumors that have re-entered the cell cycle are also less permissive to leukocyte entry. Perhaps due to the reported low rate of TEC proliferation in human breast cancers, examination of publicly available human scRNA-seq data did not reveal a clear inverse association between TEC proliferation and expression of CAMs/chemokines; however, these data did reveal that human TECs express low constitutive levels of Th1 chemokines in general^57^. Similar to murine ECs, our in vitro data using human ECs confirm low constitutive expression of these chemokines that can be induced by cytokine stimulation and further potentiated by inhibiting DNMT1. Despite our failure to clearly identify subpopulations in human tumors that matched our observations in mice, our data are in good accord with a recent study identifying *Top2a*^*hi*^ proliferating clusters of murine lung TECs that are distinct from an IFN response subpopulation enriched in both *Cxcl9* and *Cxcl10* ^46^.

Blocking DNMT1 partially mitigated FGF2’s antagonistic effect on TNFα-stimulation of CAMs/chemokines suggesting that FGF2 operates through secondary, non-redundant pathways to inhibit CAM/chemokine expression in ECs. FGF2 is a potent EC mitogen, and there are established links between cell cycle inhibition and augmentation of anti-tumor immunity. For example, CDK4/6 inhibition was shown to enhance antigen presentation, which accompanied an increased IFNγ response; interestingly, the authors linked this outcome with downregulation of DNMT1, hypomethylation of TEs, and enhanced chemokine secretion that stimulated tumor immune surveillance^5^. As predicted, the addition of ICB with CDK4/6 and PI3Kalpha inhibitors enhances anti-tumor immune responses, further supporting the concept that combinatorial approaches targeting both the cell cycle and pathways that augment T-cell function are synergistic^58^. It is possible, therefore, that cell cycle activation by EC mitogens such as FGF2 actively suppress factors in the endothelium that are important for immune surveillance by antagonizing TNFα- or IFNγ-driven pathways; thus, overcoming this immune suppressive effect of FGF2 may be needed to enhance lymphocyte entry across the endothelium into tumors.

Some limitations to this study include the possibility that, given the global nature of *Dnmt1* deletion in *Cdh5-Cre* mice, it is expected that loss of *Dnmt1* function throughout the vasculature could induce expression of CAMs and chemokines that drive immune cell infiltration into normal tissue and organ microenvironments. However, our data suggest that *Dnmt1* silencing on its own does very little to increase the constitutive expression of CAMs or chemokines; instead, *Dnmt1* inhibition appears to function more as a potentiator of CAM/chemokine expression in the presence of cytokines such as TNFα or IFNγ. Since these cytokines are expected to be enriched in the TME, this could account for a localized impact on immune/vascular cross-talk when *Dnmt1* is ablated. Furthermore, inhibiting NFκΒ or JAK2 in parallel to cytokine stimulation only partially mitigated the effect of *Dnmt1* silencing on the potentiation of CAM or chemokine expression. These results suggest that additional mechanisms, including the unmasking of immunogenic endogenous retroviruses after prolonged *Dnmt1* inhibition and potentiation of an innate IFNγ response or direct inhibition of proliferation, could be operative in our in vivo models^2,5,8,59^. Finally, while the present study has focused on how *Dnmt1* deletion in ECs impacts the total numbers of CD8^+^ T-cells present in the TME, future studies could focus on how chemokines (e.g., Cxcl9/10) or other factors expressed by ECs in the context of *Dnmt1* deletion have direct angiostatic effects that tandemly regulate T-cell exhaustion or T-cell intra-tumoral motility and survival.

Combinations of immunomodulatory drugs such as Durvalumab or Pembrolizumab, and hypomethylating agents including 5-Aza and Decitabine are currently being tested in clinical trials, but these studies are far from complete (NCT02811497 and NCT02957968). It is possible that any benefit from these combinatorial approaches could be due to impacts on cancer cells or immune cells directly in addition to epigenetic reprogramming of the endothelium. This could result in an activated tumor vasculature that (i) guides lymphocytes towards the TME (ii) is more receptive to capturing anti-tumor immune cells, and (iii) facilitates diapedesis to overcome immune exclusion and boost anti-tumor immunity.

## Methods

All animal experiments were carried out in accordance with and under the approval of the Institutional Animal Care and use Committee (IACUC) of the University of Virginia (accredited by AAALAC International) and followed the Public Health Service Policy for the Care and Use of Laboratory Animals. Animal care was provided in accordance with the procedures outlined in the Guide for the Care and Use of Laboratory Animals, and protocols were approved by IACUC (Protocol #4146). The maximum tumor size of 1 cm^3^ was permitted by IACUC. In some cases, this limit has been exceeded by the last day of measurement and the mice were immediately euthanized.

### Reagents and cell lines

Antibodies used in this study were anti-DNMT1 (Abcam), anti-5mC (Cell Signaling), anti-GAPDH, anti-ERK, anti-phospho-ERK (Cell signaling), anti-SMA (Sigma), anti-CD8 (Biolegend, BioXcell, or eBiosciences), anti-CD11c, anti-CD45, anti-F480, anti-MECA-79, anti-Vcam1, and anti-CD4 (all from BD Biosciences), anti-granzyme B (Cell Signaling), anti-mouse IgG (BioXcell), anti-PD-1 (CD279, BioXcell), and anti-PD-L1 (Genentech, MTA program). MGECs were isolated and cultured as described previously by our lab^60,61^. Human umbilical vein endothelial cells (HUVEC) were purchased from Lonza and cultured in EBM-2 supplemented with the EGM-2 bullet kit. Culture dishes were precoated with 1% gelatin. For the labeling of CD8^+^ T-cells, CellTracker^TM^ green dye was used (Molecular Probes). The culture of EO771 and PyMT cells was described previously^62,63^. Other reagents include a JAK2 inhibitor (AG490), NFκΒ inhibitor (JSH23, Sigma), TNFα (Peprotech), IFNγ (EMD Millipore), GSK3484862 (Med Chem Express), and 5-Azacytidine (Sigma).

### Animal models

The following mouse strains were used (all on a C57BL/6 background): *Cdh5*^CreERT2^:ZsGreen^l/s/l^ mice (control mice; Tg*Cdh5*-Cre/ERT2^1Rha^) and *Cdh5*^CreERT2^:*Dnmt1*^fl/fl^:ZsGreen^l/s/l^ mice (*Dnmt1*^iECKO^); *Dnmt1*^fl/fl^ mice were a gift from Dr. Muthusamy Thangaraju at Georgia Regents University. Postn^CreERT2^ mice and ZsGreen^l/s/l^ (Ai6) mice were purchased from Jackson Labs. Female mice aged 8 weeks of age were used for all experiments since mammary tumor models are used throughout this manuscript. All mice were bred and maintained in the University of Virginia animal facility in accordance with the guidelines of IACUC.

### Transfection of endothelial cells with siRNA oligos

For the siRNA transfection of ECs, control siRNA or *Dnmt1* siRNA (BLOCK-iT siRNA oligo; Invitrogen) were mixed with RNAi Max (Thermo Fisher Scientific) and incubated for four hrs in Opti-MEM-reduced serum medium (Thermo Fisher Scientific).

### Real-time polymerase chain reaction measurement of CAMs and chemokines

RNA was extracted from tissues or cell cultures using an RNA purification kit (Zymo Research). cDNA synthesis was carried out using an iScript synthesis kit (Bio-Rad). Quantitative Reverse Transcription PCR RNA was used for qRT-PCR using SYBR Green (Invitrogen). Results were analyzed via the ΔΔCt method and normalized to the *Gapdh* housekeeping gene. All primer sets are included in Supplementary Data 1. For the TNFα or IFNγ treatments, cells were cultured in growth media w/o angiogenic factors. HUVECs (passage five or six) were seeded at a density of 2 × 10^5^ cells. After 24 hrs, the HUVEC growth medium was switched to an angiogenic factor-depleted medium. Five azacytidine was added for 48 hrs., and then cells were treated with TNFα (10 ng/mL) or IFNγ (1000 units) for three hrs.

### EC adhesion assay and trans-well assays

Murine naive CD8^+^ T-cells were freshly isolated from the spleens of C57BL/6 mice using PE-anti-mouse CD8 antibody and anti-PE-conjugated magnetic beads (Miltenyi). To activate CD8^+^ T-cells, they were incubated with CD3/CD28 Dynabeads in lymphocyte medium (RPMI 1640, 10% FBS, 1% MEM NEAA, 1% 0.5 M HEPES buffer, 1% L-glutamine, 1% sodium pyruvate and 0.0004% βM-EtOH). CellTracker^TM^ green was used for labeling CD8^+^ T-cells. For adhesion assays, 5 × 10^4^ ECs were plated in 24-well plates and then treated with *Dnmt1* siRNA. Next, TNFα or IFNγ was added for 6 hrs, and then 1 × 10^6^ activated CD8^+^ T-cells were plated on the EC monolayers and incubated for one hr. After washing out the unbound cells, plates were imaged with an inverted fluorescence microscope. The average numbers of bound cells from >3 separate fields per well were quantified. For trans-well assays, ECs with and without *Dnmt1* siRNA were plated on the upper and bottom chambers. TNFα or IFNγ was added in the bottom chamber. CellTracker^TM^ green-labeled CD8^+^ T-cells (1 × 10^5^ cells), activated as above, were incubated with IgG or CXCR3 antibodies and then seeded into trans-well inserts which contained polycarbonate membranes of a 5 μm pore size (Costar) and left to migrate through EC monolayers for 90 mins. Trans-wells were fixed with 4% paraformaldehyde, and trans-migrated T-cells were counted under the microscope from at least three separate fields.

### Cytokine protein array analyses

Cell culture supernatants were concentrated with Microsep™ Advance Centrifugal Devices (PALL Corporation) and analyzed with a mouse ProteomeProfiler^TM^ array kit by following the manufacturer’s instructions (R&D).

### Luminex assays

Concentrated conditioned media was coupled to Luminex magnetic microspheres using a Luminex coupling kit (mouse Luminex panel) following the manufacturer’s instructions. The samples were analyzed using a Luminex MAGPIX (Luminex) by the UVA Flow Cytometry Core Facility.

### Nuclear fraction preparation and phos-tag immunoblotting

Cells were rinsed with cold PBS and then scraped in cold buffer A (250 mM sucrose, 50 mM Tris–HCl pH 7.4, 5 mM MgCl_2_, protease, and phosphatase inhibitors). Following centrifugation at 200 × *g* for 10 mins at 4 °C, the supernatant was used for subsequent isolation of cytosolic fractions. The pellet was resuspended in buffer A. After incubation on ice for 30 mins and centrifugation at 800 × *g* for 15 mins at 4 °C, the pellet was resuspended in buffer A and centrifuged at 500 × *g* for 15 mins. This fraction was washed and centrifuged at 1000 × *g* for 15 mins. The pellet was then dissolved in buffer B (20 mM HEPES pH 7.9, 1.5 M MgCl_2_, 0.5 M NaCl, 0.2 mM EDTA, 20% glycerol, 1% Triton-X-100, and protease and phosphatase inhibitors). The pellet was gently resuspended, vortexed, and then incubated on ice for 30 mins. After incubation on ice, the nuclear fraction was sonicated at a high setting for 15 secs with a 30-sec pause. The lysate was centrifuged at 9000 × *g* for 30 mins at 4 °C. The resultant supernatant was the final nuclear fraction. For phosphorylated DNMT1-detection, cytosolic and nuclear fractions were subjected to analysis using SuperSep^TM^Phos-tag^TM^ following the manufacturer’s instructions (Fujifilm).

### Immunohistochemistry

Harvested tumors or tissues were placed in 4% PFA in PBS for 24 hrs at 4 °C. Tumors were then transferred to a 30% sucrose solution for cryoprotection. Afterwards, tumors were embedded in OCT. Serial tissue sections were made at −20 °C using a sliding microtome. Sections were washed in PBS/0.1% Triton X-100 prior to blocking with 5% normal goat serum and 4% bovine serum albumin in PBS/0.1% Triton X-100 for one hr. Primary antibodies were applied overnight. After washing in PBS/Triton X-100, the secondary antibody was applied for two hrs at room temperature. After nuclei staining with DAPI (Molecular Probes) for 30 min, sections were mounted in Vectashield^TM^ (Molecular Probes). For vascular permeability, tumor-bearing mice received an intravenous administration of 100 μl of Tetramethylrhodamine isothiocyanate-Dextran (70 KDa, Sigma-Aldrich) using 5 mg per mouse. Tumors were harvested after 20 mins, fixed in paraformaldehyde, and transferred to sucrose. The tumor was embedded in OCT and sectioned at 7 μm. Images were collected for ZsGreen at 500–550 nm, TRITC-dextran at 555–625 nm, and DAPI at 352–402 nm. The fluorescent intensity was quantified by NIS Elements software (version 5.11.02, Nikon).

### Image processing and morphological analysis

Blood vessel analysis was performed using Fiji software by applying a threshold transformation that maximizes the global average contrast of edges. Once binary images were obtained, images were processed as follows: blood vessel structure was first extracted from the background. The skeletonization feature was then applied to binary images. The length of the blood vessel branches and branch numbers were then determined. For pericyte coverage analysis, SMA^+^ and ZsGreen^+^ cells were quantified from multiple microscopic fields from *n* = 3 tumors and using NIS Elements software (Nikon).

### Generation of EO771-expressing mCherry^+^ cell line

The pLV-mCherry reporter was produced by co-transfection into HEK293T cells with pMD2.G and psPAX2 constructs using Lipofectamine 2000 transfection reagent. Lentiviral supernatants were harvested at 72 hrs post-transfection and filtered through a 0.45 μm membrane. Cells were infected for 48 hrs with fresh lentivirus with 8 μg/mL polybrene. The cells were then selected for puromycin resistance (7.5 μg/mL) for one week and maintained in a medium containing Blasticidin. Finally, the expression of mCherry was validated using FACS.

### Sprouting angiogenesis assay

The in vitro angiogenesis sprouting assay was carried out using well-established methodologies previously described^64^. Vessel sprout numbers were quantified from multiple wells (*n* = *3*) using NIS Elements software (Nikon).

### In vitro microvascular networks

Vascular networks were created in microfluidic devices by mixing human umbilical vein endothelial cells (HUVEC) and human lung fibroblasts with fibrinogen and thrombin before injecting them into the central channel of microfluidic devices (AIM Biotech). The final concentration of fibrin was 2.8 mg/ml with 8 × 10^6^/mL HUVEC and 2 × 10^6^/mL fibroblasts. After fibrin polymerization, luer connecters were added to the media reservoirs and filled with Vasculife medium (Lifeline Cell Technology) such that a 2 cm height difference was created to drive interstitial flow across the gel. ECs self-assembled into microvascular networks, and media was changed daily to re-establish the pressure differential across the gel. After six days, microvascular networks were treated with 1 μM 5-Aza for 24 hrs, followed by treatment with IFNγ or TNFα for 24 hrs. Jurkat T cells overexpressing CXCR3 were dyed with CellTracker^TM^ green CMFDA, and 3 × 10^4^ cells were perfused through the microvascular networks for 30 mins^65,66^. Then, unbound cells were washed out, and networks were dyed with rhodamine UEA lectin (Vector Laboratories). Images were acquired with confocal microscopy at 10×, and the number of Jurkat T cells retained in the microvascular networks was quantified using ImageJ.

### Chromatin immunoprecipitation analysis

ECs were treated with TNFα, IFNγ, or FGF2 for 24 hrs, fixed with 1% formaldehyde, and lysed. Chromatin was subjected to shearing by sonication. Immunoprecipitations were prepared using antibodies for murine DNMT1. The precipitated chromatin was then captured by protein A or G magnetic beads and eluted with elution buffer. After chromatin immunoprecipitation, DNA was purified by phenol/chloroform extraction. Immunoprecipitated chromatin was used for real-time qPCR amplified with primers directed to promoter regions of murine *Cxcl9* or *Cxcl10*.

### Flow cytometry

Tumors or normal tissues from control mice or *Dnmt1*^iECKO^ mice were minced into small pieces and incubated for 90 mins at 37 °C in dissociation buffer containing 2 mg/mL of collagenase type I (Worthington), 1 mg/mL of DNase (Worthington), and 2.5 units/mL of neural protease (Worthington). The digested tissues were separated into a single-cell suspension by passing through a 100 μM cell strainer. Single-cell isolates were stained with primary antibodies for 30 mins on ice, followed by secondary antibodies, and then washed using FACS buffer. Samples were fixed with 2 % paraformaldehyde and then analyzed by flow cytometry on a FACSCaliber (BD) or Accuri C6. In some experiments, single-cell isolates were incubated with CD45-APC and CD31-PE antibodies and then sorted to obtain live CD45^-^/CD31^+^ ECs. These isolated ECs were then subjected to RNA purification for qPCR. All FACS data was post-analyzed using FlowJo software (Tree Star Inc.) for quantification of specific cell populations.

### In vivo primary tumor models

For primary tumor growth, mice were orthotopically injected with 5 × 10^5^ EO771 or PyMT mammary tumor cells resuspended in 100 μL HBSS into the 4th left mammary fat pad under anesthetic (5% ketamine; 1% xylazine solution). For immune checkpoint inhibition, when tumors reached a volume of ~100 mm^3^, animals were injected with IgG, anti-CD8a (20 mg/kg, clone 2.43, BioXCell), (anti-PD-1, 15 mg/kg, clone 2A3, BioXCell), anti-PD-L1 (10 mg/kg, Genentech MTA program), or anti-CTLA-4 (5 mg/kg, 9H10, BioXCell) antibodies every three days for a total of three treatments. Tumor growth was monitored each day using calipers. Tumors were harvested when they reached ~1 cm^3^ in size. Tumor volumes were calculated using the modified ellipsoid formula; 1/2 × (length × width^2^). For experimental lung metastasis studies, 1 × 10^5^ of EO771^mCherry^ cells in 100 μL HBSS were injected via the tail vein. After two weeks, the mice were euthanized, and the lungs were harvested for cryosections and immunohistology.

### Single-cell RNAseq

For EC sorting, tissues were minced into small pieces and added to a GentleMACs C tube (Miltenyi) and dissociated using the Miltenyi Tissue Dissociator (Miltenyi). The homogenized tissues were incubated for 60 min at 37 °C in a dissociation buffer containing 2 mg/ml of collagenase type I (Worthington), 1 mg /ml of DNase (Worthington), and 2.5 units/ml of neural protease (Worthington). The digested tissues were then separated into a single-cell suspension by passing through a 100 μM cell strainer. The cell pellet was washed three times at 1200 rpm for 5 min and resuspended in Pharmlyse B (BD Pharmingen) for 5 mins at room temperature. After removing the Pharmlyse B by centrifugation, cells were washed in FACS buffer and then stained with Live dye (Live-or-Dye™ Fixable Viability Staining Kits, Biotium). For Fc blocking, cells were incubated with mouse FcR blocking reagent (Miltenyi) for 10 min. Cells were then stained with PE-conjugated CD31 (1:50) and APC-conjugated CD45 antibodies (1:50) (BD Pharmingen) for 30 min on ice. After incubation, cells were washed three times at 1200 rpm for 5 min and then sorted by an Influx Cell Sorter (Becton Dickinson).

After library preparation, the FASTQ files were mapped using the CellRanger pipeline (10X Genomics) to generate a count matrix. The matrix generated by the CellRanger pipeline was imported into the Seurat R package for performing the additional QC and in-depth analysis. In summary, cells that had low RNA counts (<200 genes) were filtered out. The cells were also filtered based on <10% mitochondrial genes. The data was normalized after removing unwanted cells (immune cells, pericytes, smooth muscle cells, and epithelial cells) from the dataset by employing a global-scaling normalization method, “LogNormalize” that normalizes the feature expression measurements for each cell by the total expression. The highly variable (highly expressed in some cells and lowly expressed in others) top 4000 genes were used for downstream analysis. Random permutated subsets of the data (1% by default) were used to rerun the PCA and to construct a ‘null distribution’ of feature scores to identify the number of PCs to consider for performing the clustering analysis. The FindNeighbors and FindClusters functions were used from the Seurat object. The FindNeighbors function works by calculating the neighborhood overlap (Jaccard index) between every cell and its k.param nearest neighbors. FindClusters uses a graph-based clustering approach and a Louvain algorithm. Non-linear dimensionality reduction (UMAP/tSNE) was used to explore and visualize the clusters.

### Statistics and reproducibility

All values are expressed as mean ± standard deviation of the mean (STD). All results were analyzed by Student’s *t* test or ANOVA with multiple comparisons using GraphPad Prism software. Experiments have been repeated a total of three times or had a sufficient number of mice to detect a statistically significant difference in the means. *P* values <0.05 were considered statistically significant.

### Graphical content

Graphical content (Supplementary Figs. 1a and 13a and Figs. 3a and 7h) was created with BioRender.com.

### Reporting summary

Further information on research design is available in the Nature Portfolio Reporting Summary linked to this article.

## Supplementary information

Supplementary Information

Description of Additional Supplementary Files

Supplementary Data 1

Reporting Summary

## Data Availability

The scRNAseq data generated in this study have been deposited in the NCBI GEO database under accession code GSE186467. The publicly available scRNAseq data generated by Hua et al.^41^ have been deposited in the NCBI GEO database under accession code GSE198080. The publicly available scRNAseq data generated by Wu et al.^44^ are available at: https://zenodo.org/record/5031502#.Y_Og3i2cbUI. The publicly available scRNAseq data generated by Wu et al.^45^ are available from: [https://github.com/sunnyzwu/stromal_subclasses]. The remaining data are available within the Article, Supplementary Information, or Source Data file. Source data are provided with this paper.

## Source data

Source Data
