# Supplementary material for: Priming a vascular-selective cytokine response permits CD8+ T-cell entry into tumors
Source: Nat Commun. 2023 Apr 14;14:2122. doi: 10.1038/s41467-023-37807-z (PMC10101959; doi:10.1038/s41467-023-37807-z)
Supplement: Supplementary file 1 — Supplementary Information [file 41467_2023_37807_MOESM1_ESM.pdf]

Supplementary Information for

Priming a vascular-selective cytokine response permits CD8$^+$ T-cell entry into tumors

Dae Joong Kim, Swetha Anandh, Jamie L. Null, Piotr Przanowski, Sanchita Bhatnagar, Pankaj Kumar, Sarah E. Shelton, Erin E. Grundy, Katherine B. Chiappinelli, Roger D. Kamm, David A. Barbie, and Andrew C. Dudley*

*Corresponding author. Email: acdudley@virginia.edu

Supplementary Figures 1 to 18.

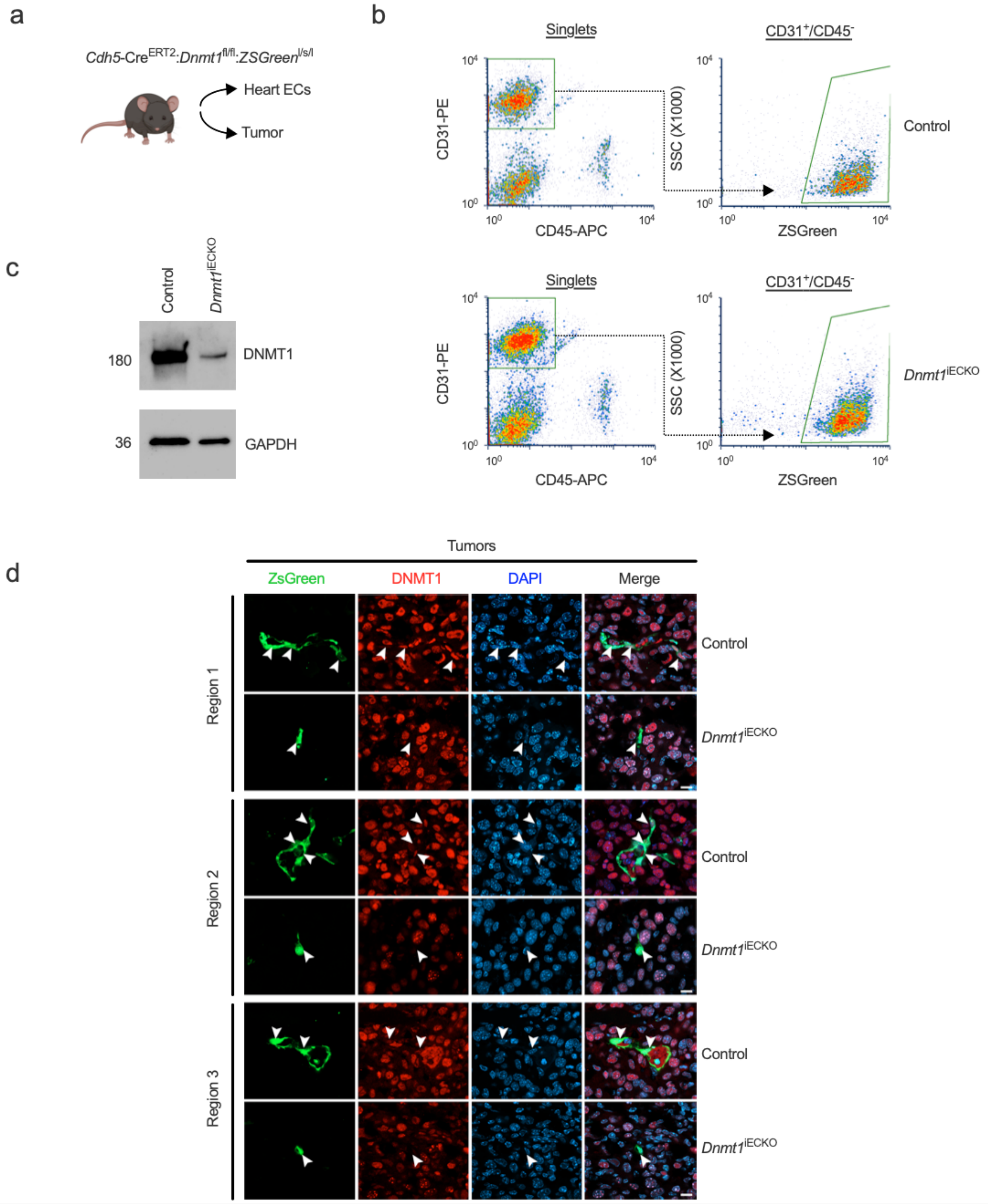

Supplementary Fig. 1.
(a) Schematic representation of EC isolation strategy. (b) FACS gating strategy for the purification of $CD45^-/CD31^+/ZsGreen^+$ populations. (c) Immunoblot analysis for DNMT1 expression in sorted EC lysates isolated from control and *Dnmt1*^{iECKO} mice

(representative of *n=2* independent blots). (d) Representative immunofluorescent images of DNMT1 (red) and ECs (ZsGreen) in tumor samples from control and *Dnmt1*^{iECKO} mice (*n=3* mice per/condition). Arrow heads point to ECs and absence of DNMT1 in ECs from *Dnmt1*^{iECKO} mice. Scale bar = 10 μm.

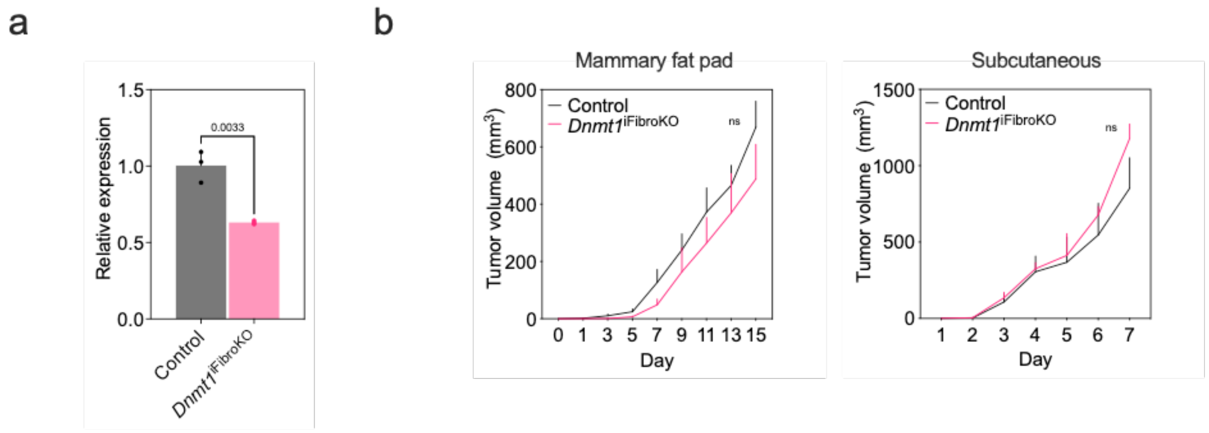

Supplementary Fig. 2.

(a) qPCR analysis for *Dnmt1* using FACS-isolated ZsGreen$^+$ cells from pooled mammary glands (*n=6*) from control versus *Dnmt1*^{iFibroKO} mice. (b) Tumor volumes determined with calipers in the indicated mice. Mammary fat pad; control, *n=5* and *Dnmt1*^{iFibroKO} mice, *n=4*. Subcutaneous, *n=3* for both groups. Data were analyzed using ANOVA followed by Sidak's multiple comparisons test. All error bars are mean -/+ STD. Source data are provided as a Source Data file.

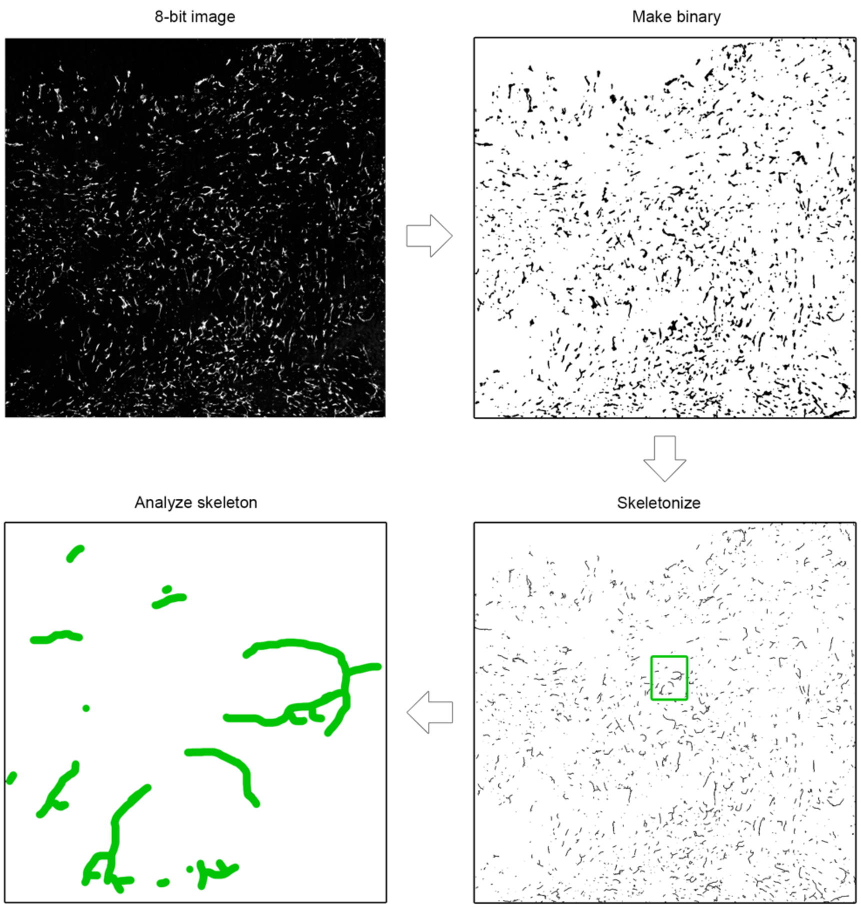

Supplementary Fig. 3.
Strategy for analyzing vessel branches in control versus *Dnmt1*^{iECKO} mice. After converting a fluorescence image into an 8-bit image, a binary image can be generated. Using the “skeletonize” plug-in from Image J then allows for unbiased, high-throughput quantification of vessel lengths and numbers of branches from whole tumor scans or regions of interest (ROIs).

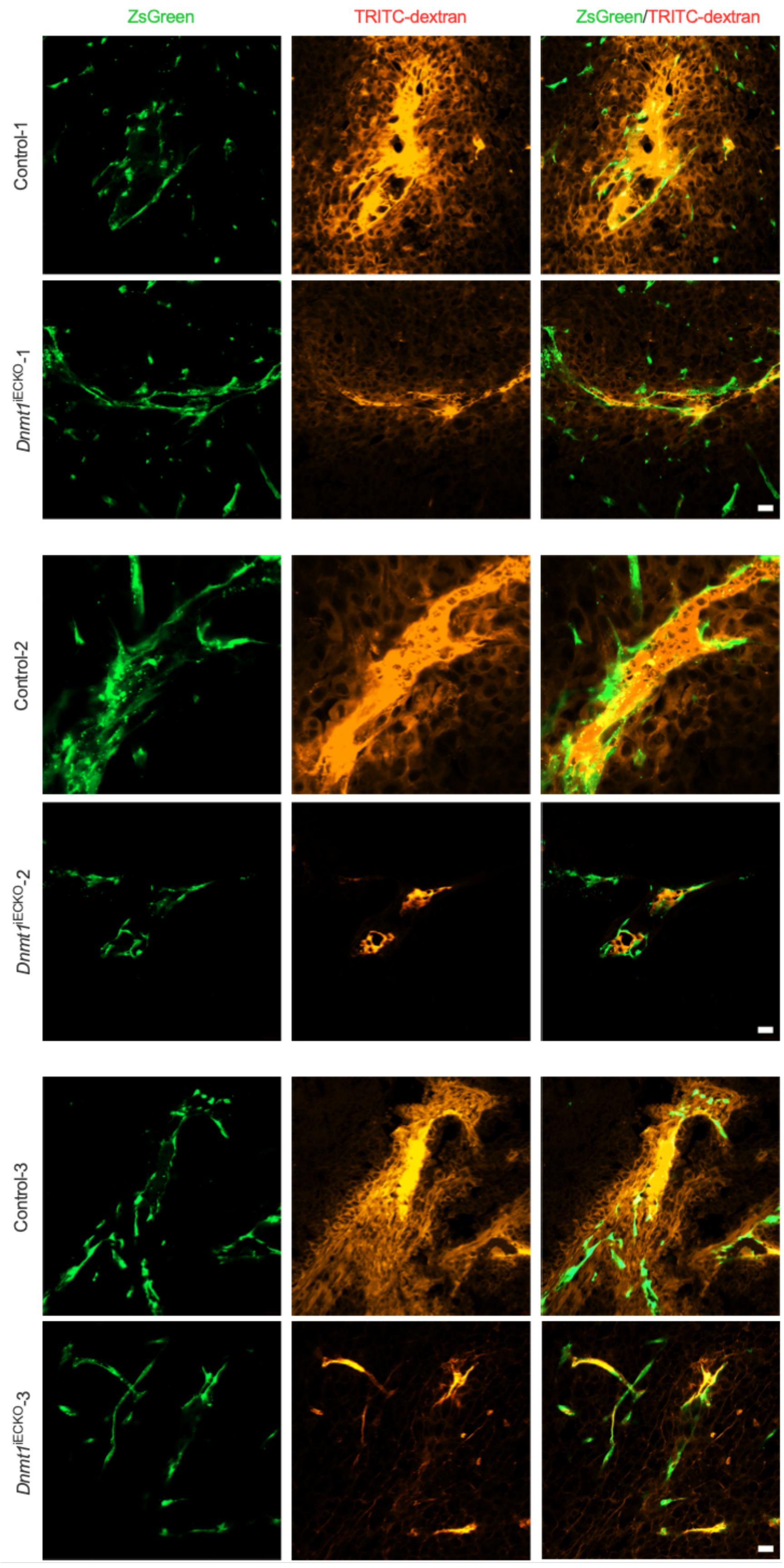
ZsGreen
TRITC-dextran
ZsGreen/TRITC-dextran
Control-1
Dnmt1iECKO-1
Control-2
Dnmt1iECKO-2
Control-3
Dnmt1iECKO-3

Supplementary Fig. 4.
Examination of an extravascular TRITC-dextran tracer injected in the tail veins of control versus *Dnmt1*^{iECKO} mice. Representative images of freshly prepared cryosections are shown (*n=3* mice/condition). Scale bar = 20 μm.

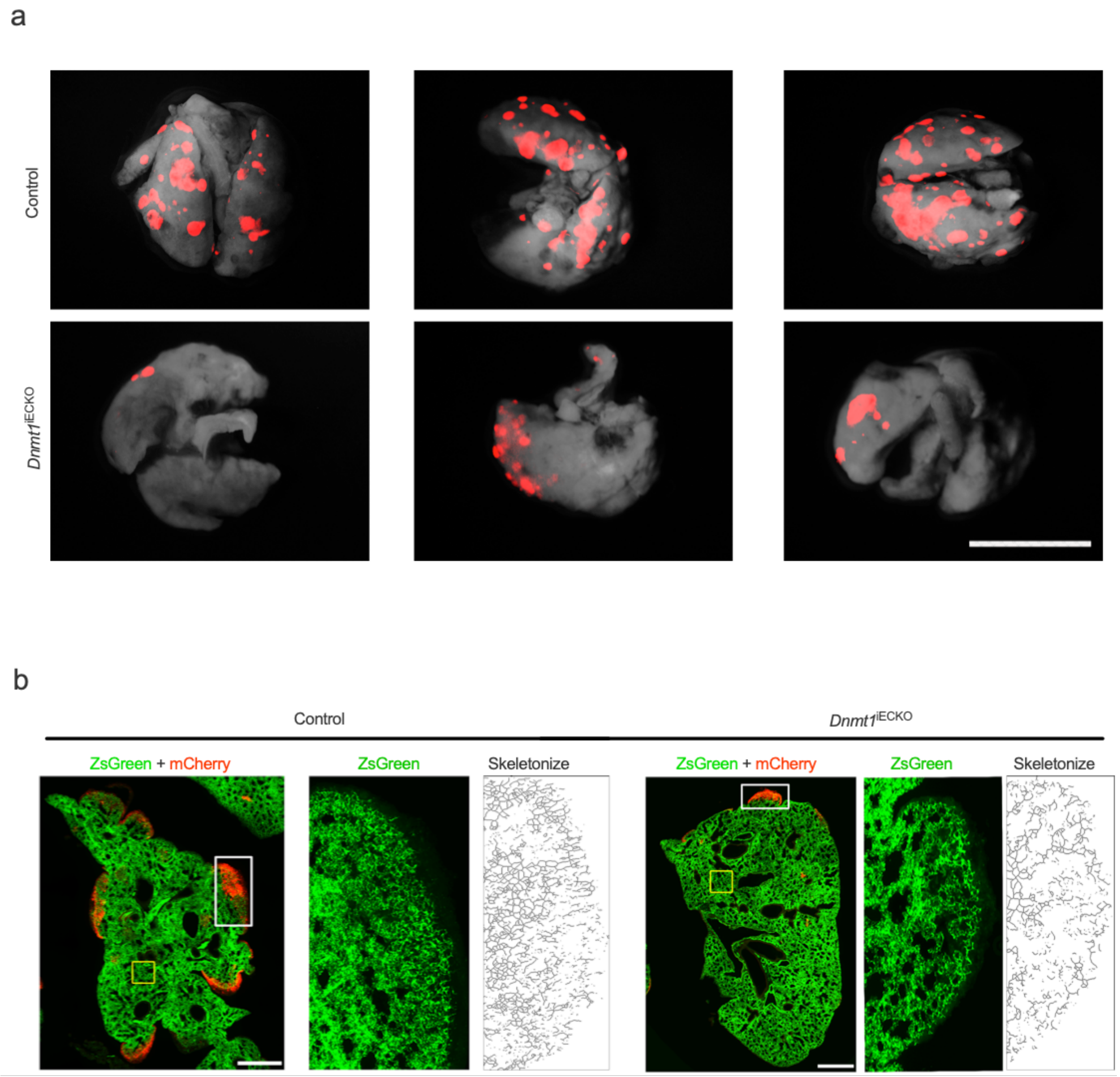

Supplementary Fig. 5.
(a) Representative images of mCherry^{+} tumor nodules in lungs from control versus *Dnmt1*^{iECKO} mice using fluorescence stereo-microscopy (*n=4* mice/condition and *n=3* mice are shown). Scale bar = 1 cm. (b) Whole image scans of lungs from control versus *Dnmt1*^{iECKO} mice following tail vein seeding of EO771^{mCherry} cells. White boxed areas (within a tumor nodule) were zoomed 5X, converted to an 8-bit binary image, and then subjected to the “skeletonize” feature in ImageJ. A representative yellow boxed area is shown that was used to assay vessel branches in normal lung regions of the same section (*n=3* individual tumors and 9 histological sections). Scale bar = 500 μm.

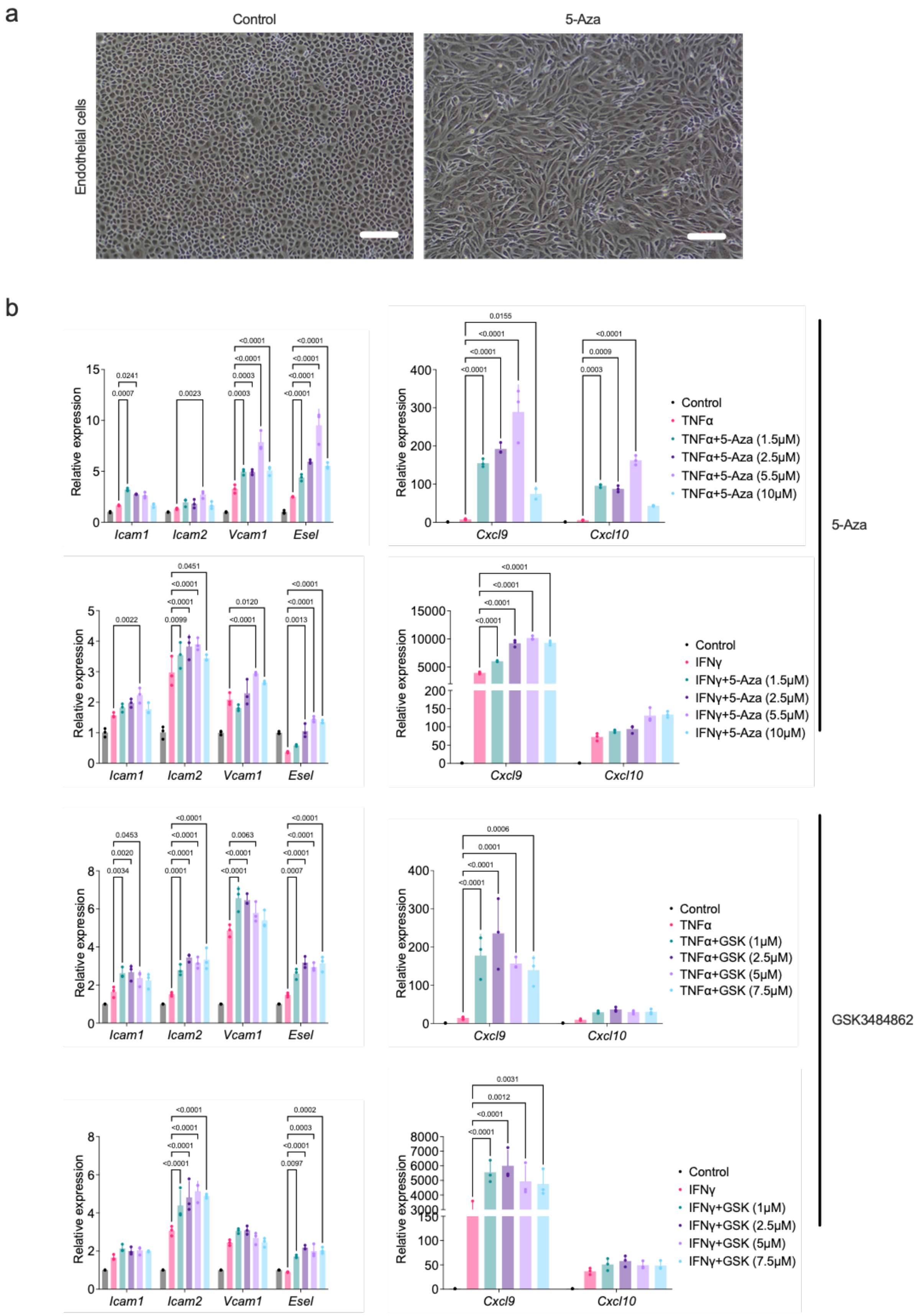
a
Control
5-Aza
Endothelial cells
b
Relative expression
Icam1
Icam2
Vcam1
Esel
Cxcl9
Cxcl10
Control
TNFα
TNFα+5-Aza (1.5μM)
TNFα+5-Aza (2.5μM)
TNFα+5-Aza (5.5μM)
TNFα+5-Aza (10μM)
IFNγ
IFNγ+5-Aza (1.5μM)
IFNγ+5-Aza (2.5μM)
IFNγ+5-Aza (5.5μM)
IFNγ+5-Aza (10μM)
5-Aza
TNFα+GSK (1μM)
TNFα+GSK (2.5μM)
TNFα+GSK (5μM)
TNFα+GSK (7.5μM)
IFNγ+GSK (1μM)
IFNγ+GSK (2.5μM)
IFNγ+GSK (5μM)
IFNγ+GSK (7.5μM)
GSK3484862

Supplementary Fig. 6.
(a) Representative morphology of MGECs treated with 5-Aza for 48 hours. Scale bar = 100 μm. (b) qPCR analysis of 5-Aza- primed or GSK3484862-primed MGECs -/+ TNFα (10 ng/mL) or IFNγ (1000U) treatment. The 5-Aza or GSK3484862 doses are shown at far right. Each data point is the mean value of three biological replicates run in triplicate and the data was analyzed using ANOVA followed by Dunnett's multiple comparisons test. Error bars are mean -/+ STD. Source data are provided as a Source Data file.

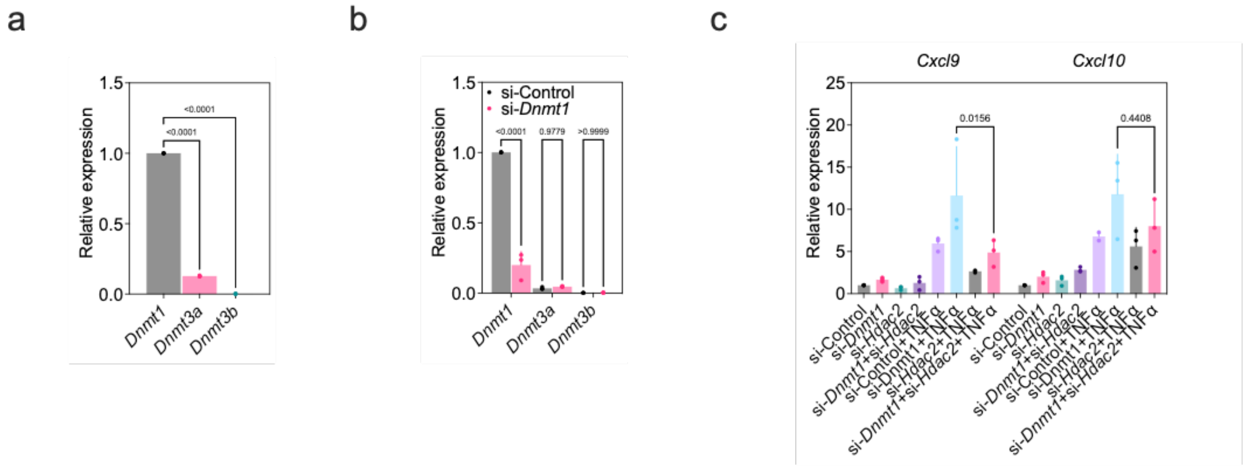

Supplementary Fig. 7.

(a) Basal expression of *Dnmt1* isoforms in MGECs determined by qPCR. Results were analyzed using ANOVA followed by Tukey's multiple comparisons test. (b) Expression of *Dnmt1* isoforms in MGEC cultures following siRNA-mediated silencing of *Dnmt1* as determined by qPCR. Results were analyzed using ANOVA followed by Sidak's multiple comparisons test. (c) qPCR analysis of *Cxcl9* or *Cxcl10* in MGECs under the indicated treatments. Results were analyzed using ANOVA followed by Tukey's multiple comparisons test. Each data point is the mean of three biological replicates run in triplicate. Error bars are mean -/+ STD. Source data are provided as a Source Data file.

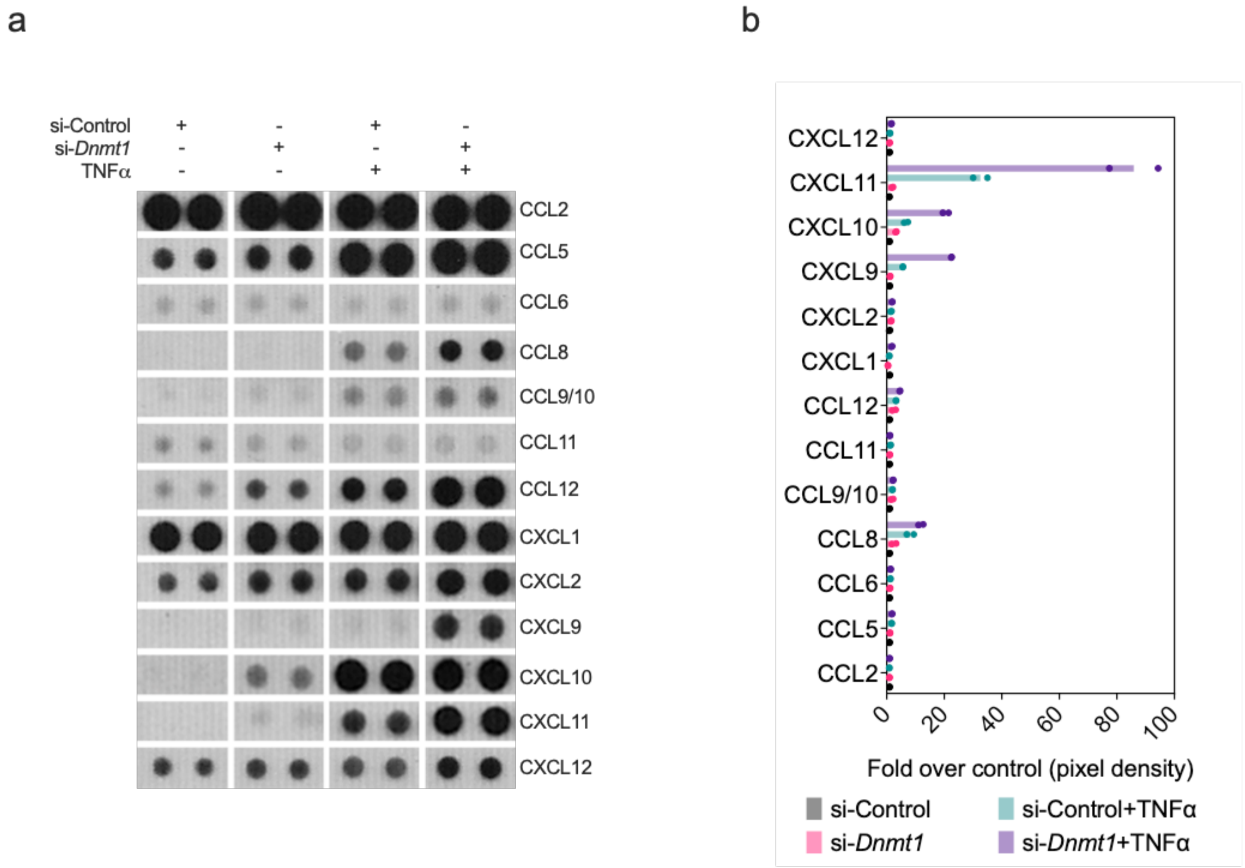

Supplementary Fig. 8.
(a) Proteome profiler array in *Dnmt1*-silenced and TNFα treated MGECs. The conditioned medium was concentrated, and then subjected to the array following the manufacturer's instructions. (b) Quantification of spot density for each detected cytokine normalized to an internal housekeeping protein on the array (note that some cytokines were not detected under any condition and have been excluded from the analysis). This experiment was repeated twice and representative results are shown. Source data are provided as a Source Data file.

a

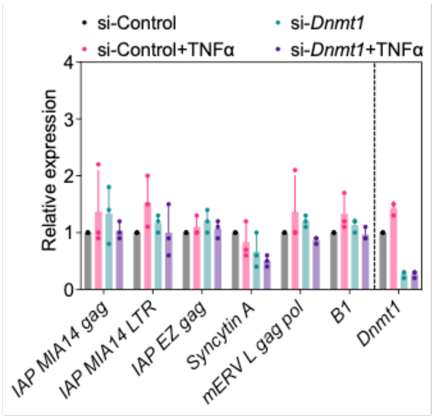

b

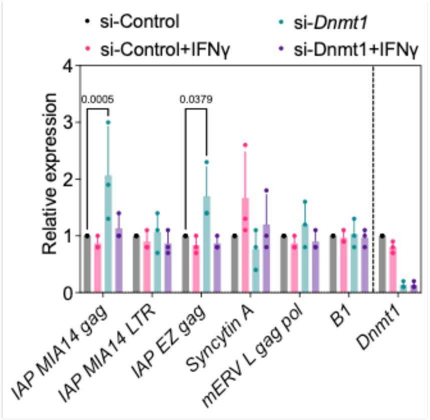

Supplementary Fig. 9.

(a) qPCR analysis of TEs in MGECs under the indicated conditions -/+ TNFα. (b) Same as in "a" except cells were treated with IFNγ. Each data point is the mean of three biological replicates run in triplicate. Results were analyzed using ANOVA followed by Dunnett's multiple comparisons test. Error bars mean -/+ STD. Source data are provided as a Source Data file.

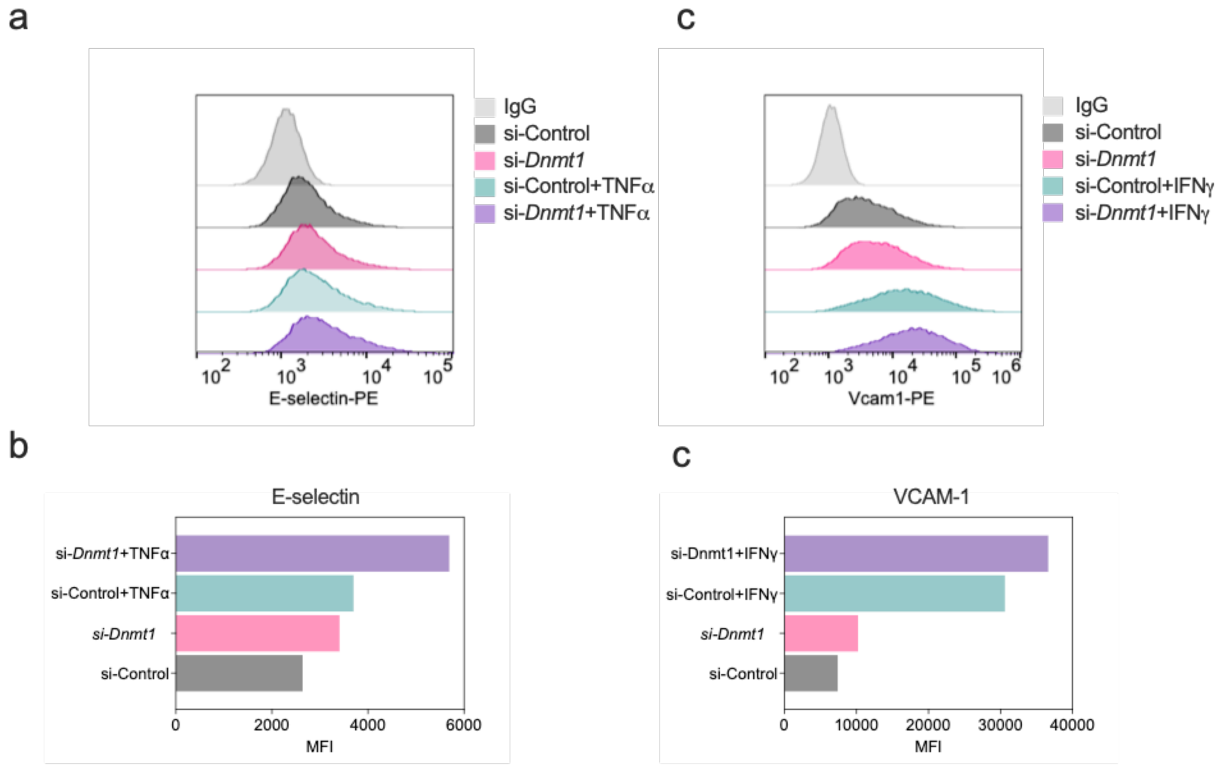

Supplementary Fig. 10.
(a) FACS plots for E-selectin surface expression in MGECs under the indicated conditions. (b) The mean fluorescence intensity (MFI) for E-selectin. (c) Representative FACS plots for VCAM-1 surface expression in MGECs under the indicated conditions. (d) The MFI for VCAM-1. Data are representative of *n=2* independent experiments. Source data are provided as a Source Data file.

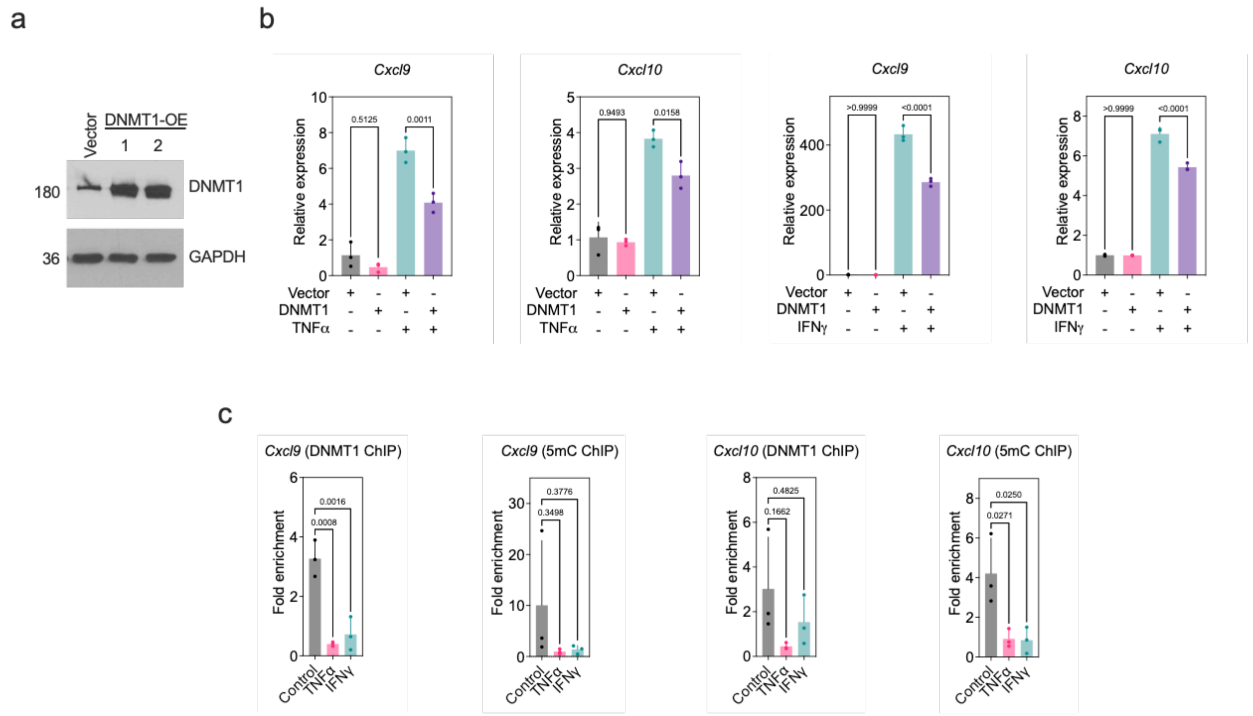

Supplementary Fig. 11.

(a) Western blot confirming DNMT1 over-expression using a pLenti construct containing full-length murine DNMT1 (representative of *n=2* independent blots). (b) qPCR analysis of *Cxcl9* and *Cxcl10* in MGEC cultures following DNMT1 over-expression alongside TNFα or IFNγ stimulation. (c) ChIP assays using the indicated capture antibody (DNMT1 or 5mC) to assess fold enrichment on *Cxcl9* or *Cxcl10* promoters. Results were generated from three biological replicates run in triplicate and analyzed using ANOVA followed by Tukey's multiple comparisons test. All error bars are mean -/+ STD. Source data are provided as a Source Data file.

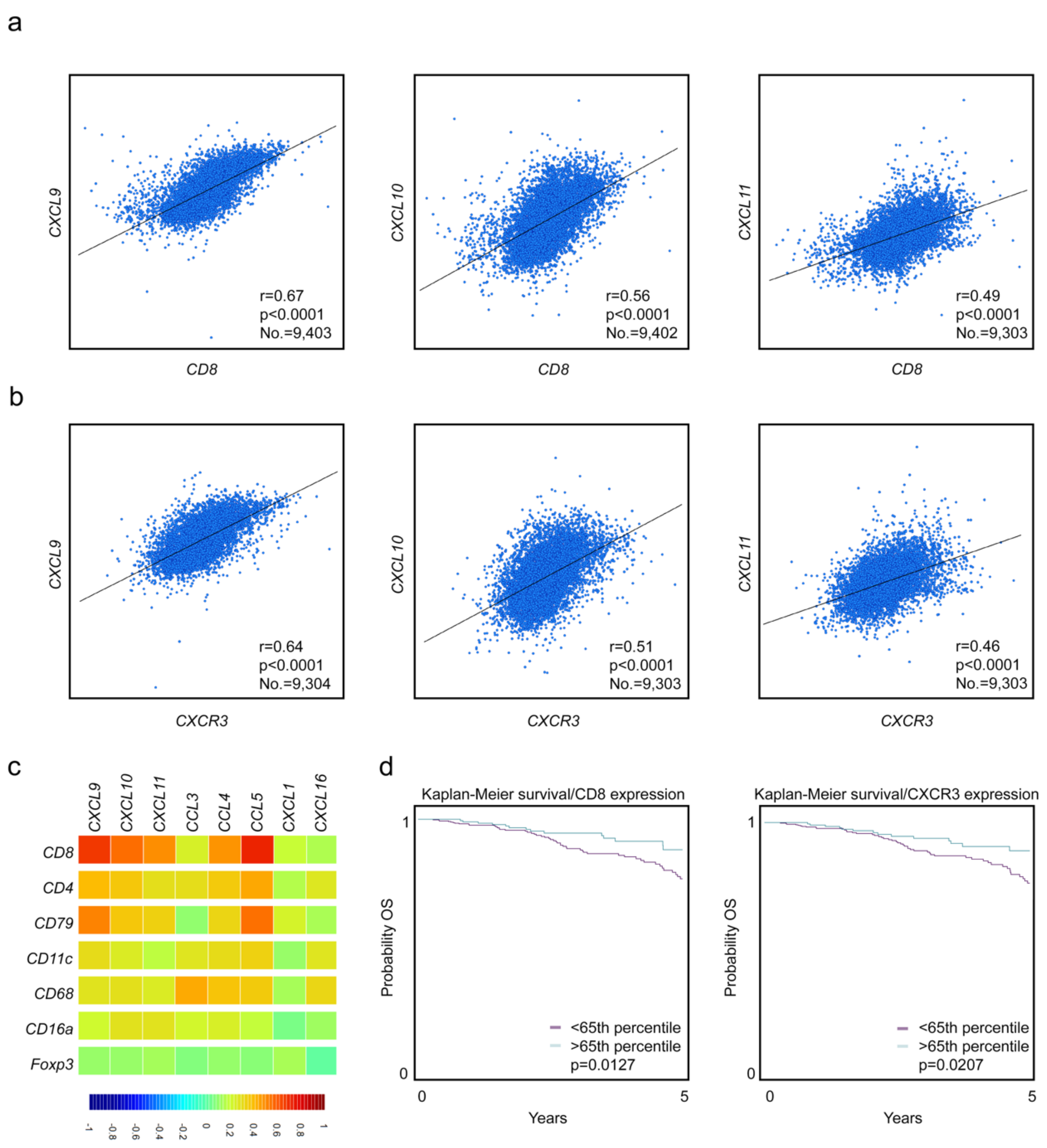

Supplementary Fig. 12.
(a) Correlations between gene expression of CD8 (a surrogate for $CD8^+$ T-cells) and the indicated chemokine. The correlation co-efficient, p-value, and number of patients are shown on each plot. (b) Same as above using CXCR3 as the marker. (c) Heat map showing associations between the indicated chemokine (top) versus the cell-selective marker. (d) Kaplan-Meier survival analysis in human breast cancers based on CD8 or CXCR3 expression used as surrogates for intratumoral T-cell content. These data were generated using the publicly available bc-GenExMiner 3.0 portal (http://bcgenex.centregauducheau.fr).

a

b

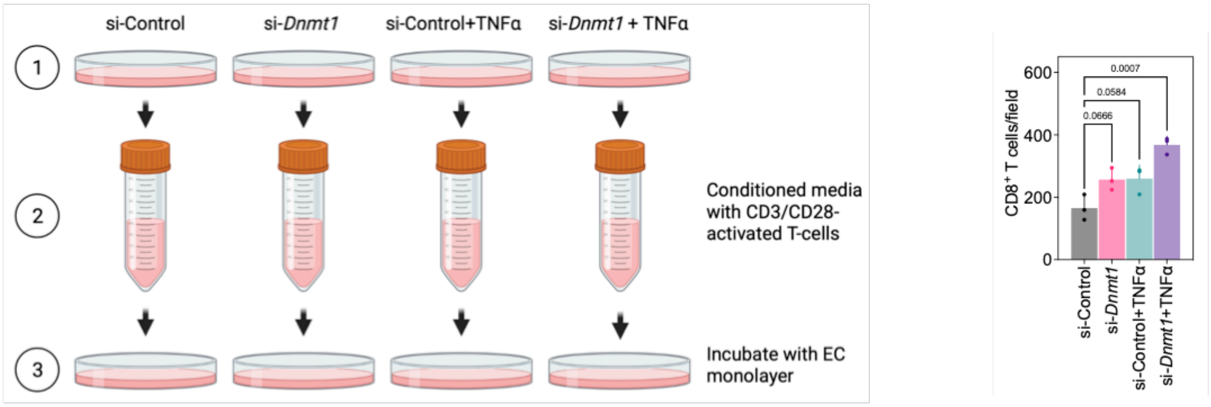

Supplementary Fig. 13.
(a) Schematic for the generation of T-cell-conditioned media with CD3/CD28-activated T-cells. The media was collected and added to MGEC monolayers. (b) Adhesion assay using the conditioned media with MGECs under the indicated conditions. Each data point represents multiple counts from a single well (*n=3* biological replicates combined in triplicate). All p-values are shown on the graph and results were analyzed using ANOVA followed by Tukey's multiple comparisons test. Error bars are mean -/+ STD. Source data are provided as a Source Data file.

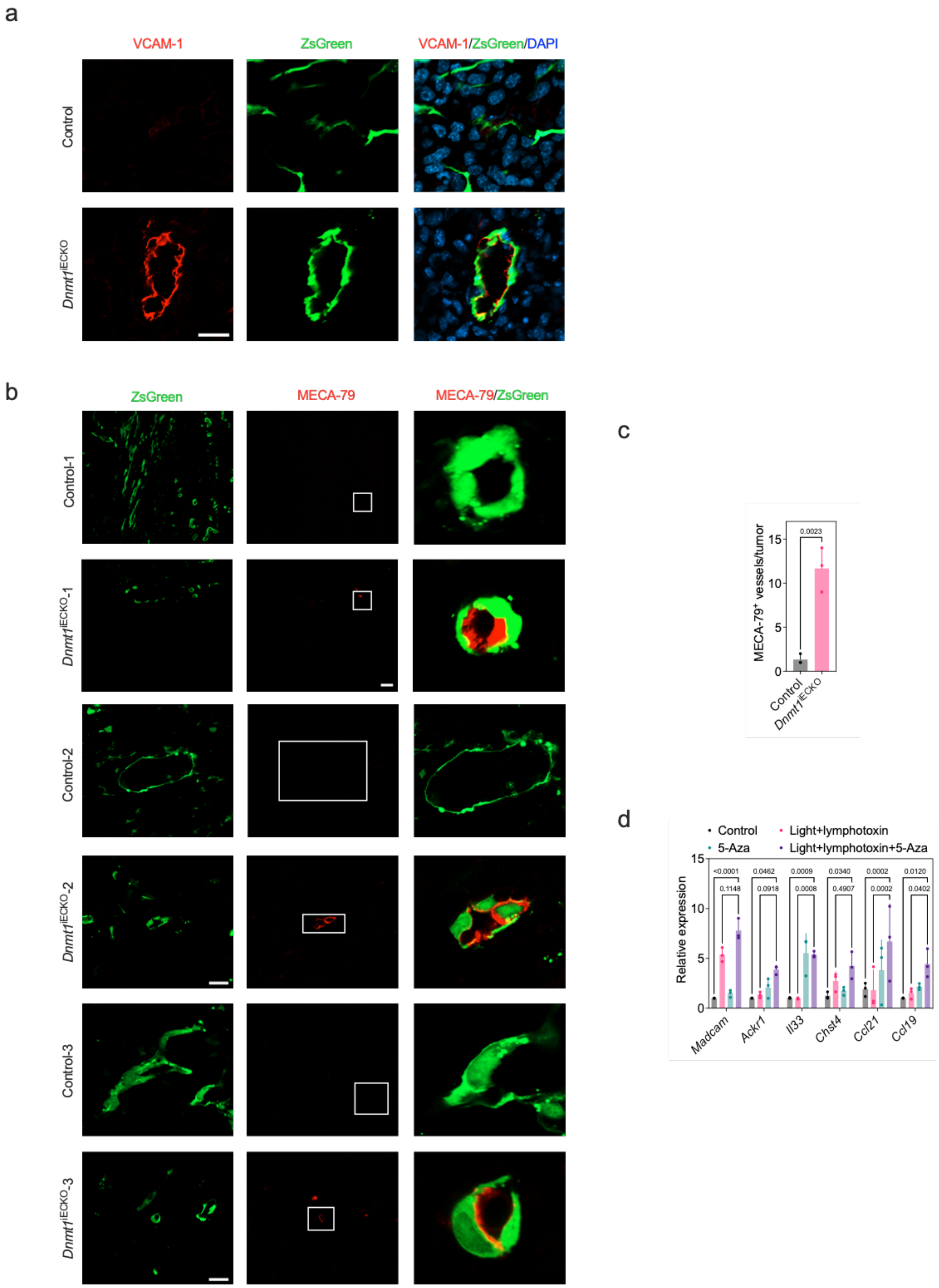

a
VCAM-1
ZsGreen
VCAM-1/ZsGreen/DAPI
Control
Dnmt1iECKO
b
ZsGreen
MECA-79
MECA-79/ZsGreen
Control-1
Dnmt1iECKO-1
Control-2
Dnmt1iECKO-2
Control-3
Dnmt1iECKO-3
c
MECA-79+ vessels/tumor
0.0023
15
10
5
0
Control
Dnmt1iECKO
d
Control
5-Aza
Light+lymphotoxin
Light+lymphotoxin+5-Aza
Relative expression
15
10
5
0
<0.0001
0.1148
0.0462
0.0918
0.0009
0.0008
0.0340
0.4907
0.0002
0.0002
0.0120
0.0402
Madcam
Ackr1
Il33
Chst4
Ccl21
Ccl19

Supplementary Fig. 14.

(a) Representative images of VCAM-1 staining in fresh cryosections using tumors from control versus *Dnmt1*^{iECKO} mice (*n=3* independent tumors/group were analyzed). Scale bar = 50 μm. (b) Multiple representative images of MECA-79 staining using tumors from the indicated mice. Boxed areas are enlarged 5X at far right to show the characteristic "plump" morphology of HEV-ECs. Scale bar = 20 μm. (c) Quantification of MECA-79^{+} vessels in the indicated mice; *n=3* mice per group). Data were analyzed using an unpaired two-tailed Student's t-test. (d) qPCR analysis for HEV marker genes in MGECs under the indicated conditions. Each data point is the mean of three biological replicates run in triplicate. Data were analyzed using ANOVA followed by Tukey's multiple comparisons test. Error bars are mean -/+ STD. Source data are provided as a Source Data file.

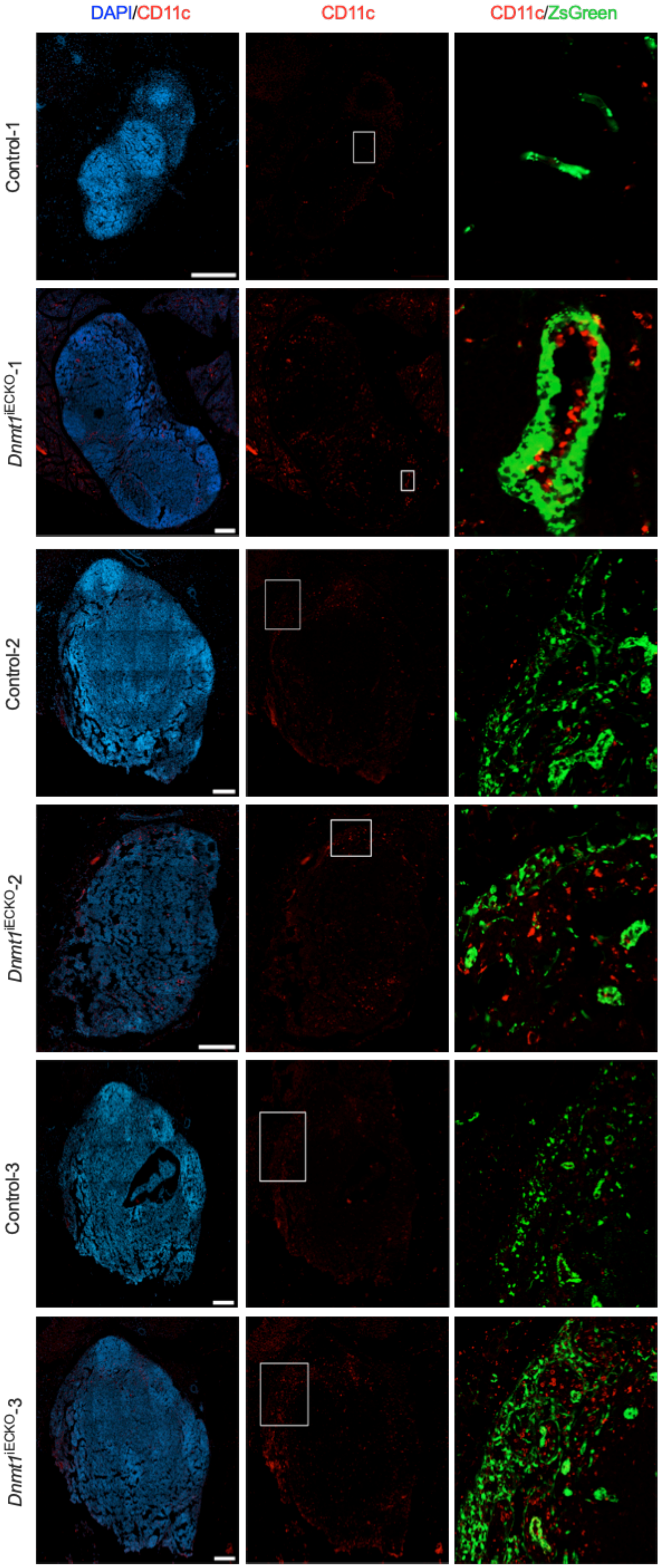
DAPI/CD11c
CD11c
CD11c/ZsGreen
Control-1
Dnmt1iECKO_1
Control-2
Dnmt1iECKO_2
Control-3
Dnmt1iECKO_3

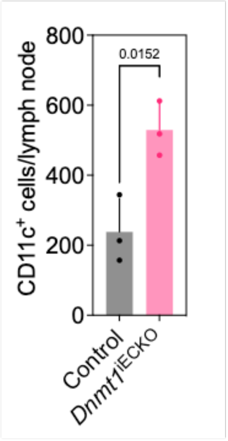
CD11c+ cells/lymph node
800
600
400
200
0
0.0152
Control
Dnmt1iECKO

Supplementary Fig. 15.

Representative images of CD11c immunohistochemistry in tumor-draining lymph nodes from *Dnmt1*^{iECKO} mice versus control mice. The boxed areas were further magnified at far right to show intravascular and perivascular CD11c^{+} cells. Scale bar = 200 μm. The graph shows the numbers of CD11c^{+} cells quantified from ROIs using individual lymph nodes (*n=3* mice per group). Data were analyzed using an unpaired two-tailed Student's t-test. Error bars are mean -/+ STD. Source data are provided as a Source Data file.

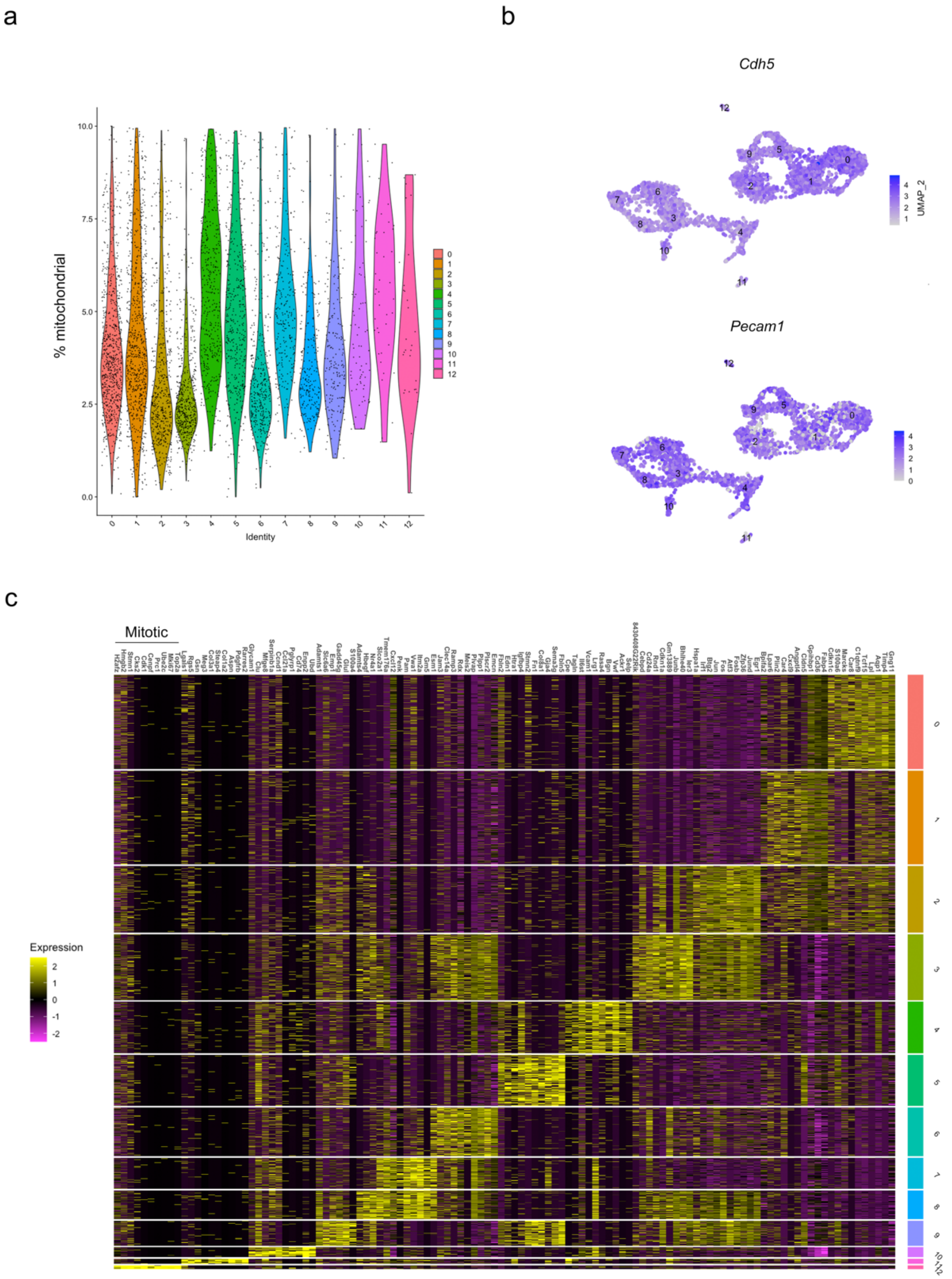

Supplementary Fig. 16.
(a) Percentage mitochondrial genes by cluster. (b) Expression of *Cdh5* and *Pecam1* amongst the different clusters. (c) Differential expression analysis showing the top 10 differentially expressed genes by cluster. The mitotic cluster is indicated on the plot.

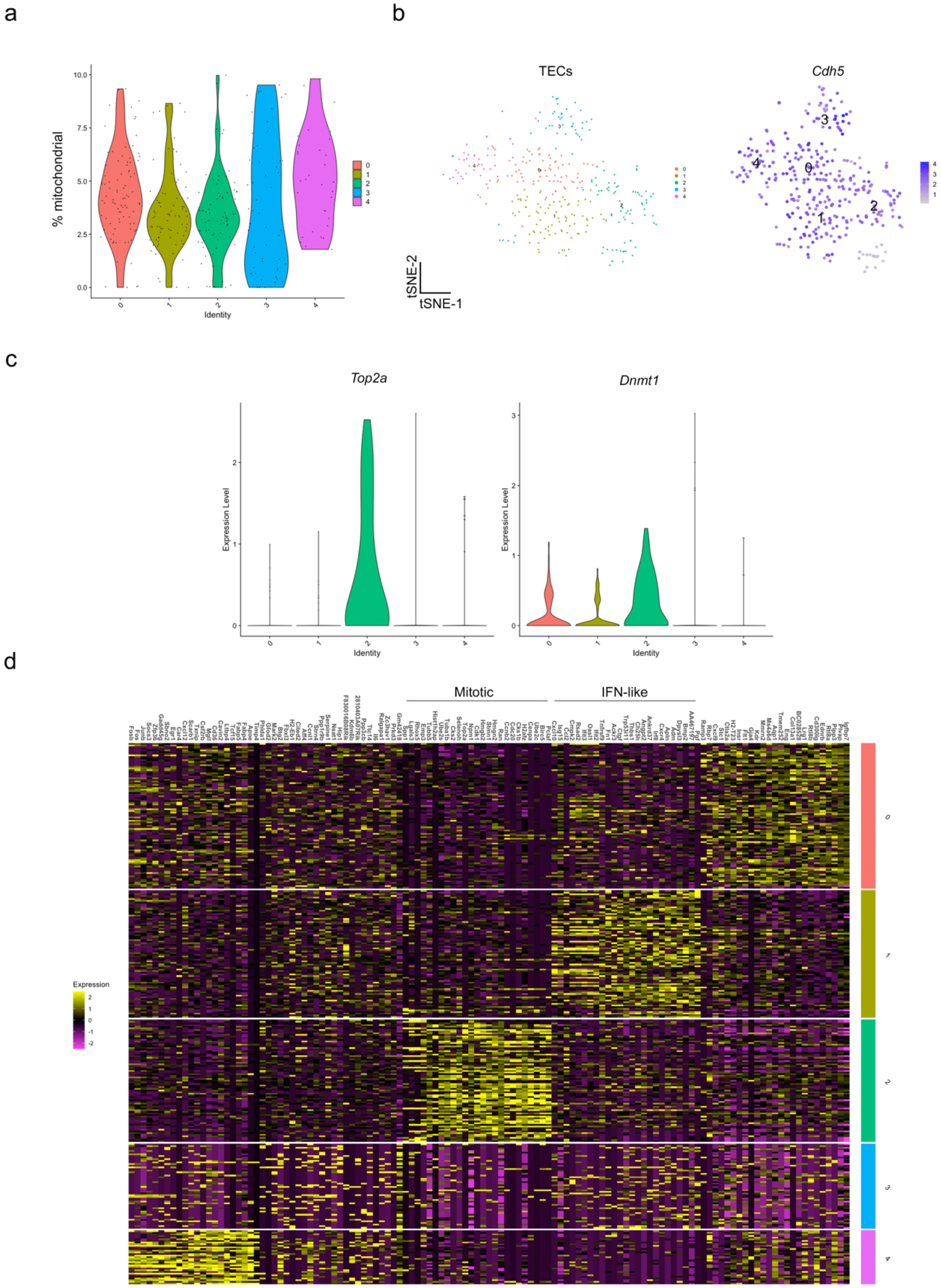

Supplementary Fig. 17.

(a) Percentage mitochondrial genes by cluster. (b) tSNE plots showing five main clusters that are positive for *Cdh5*. (c) Violin plots showing *Top2a* and *Dnmt1* expression for each cluster. (d) Differential expression analysis showing the top 25 differentially expressed genes by cluster. The mitotic cluster and the IFN-like cluster is indicated on the plot. For the data projection, NECs and TECs were first clustered together and then the TECs were separated and re-clustered.

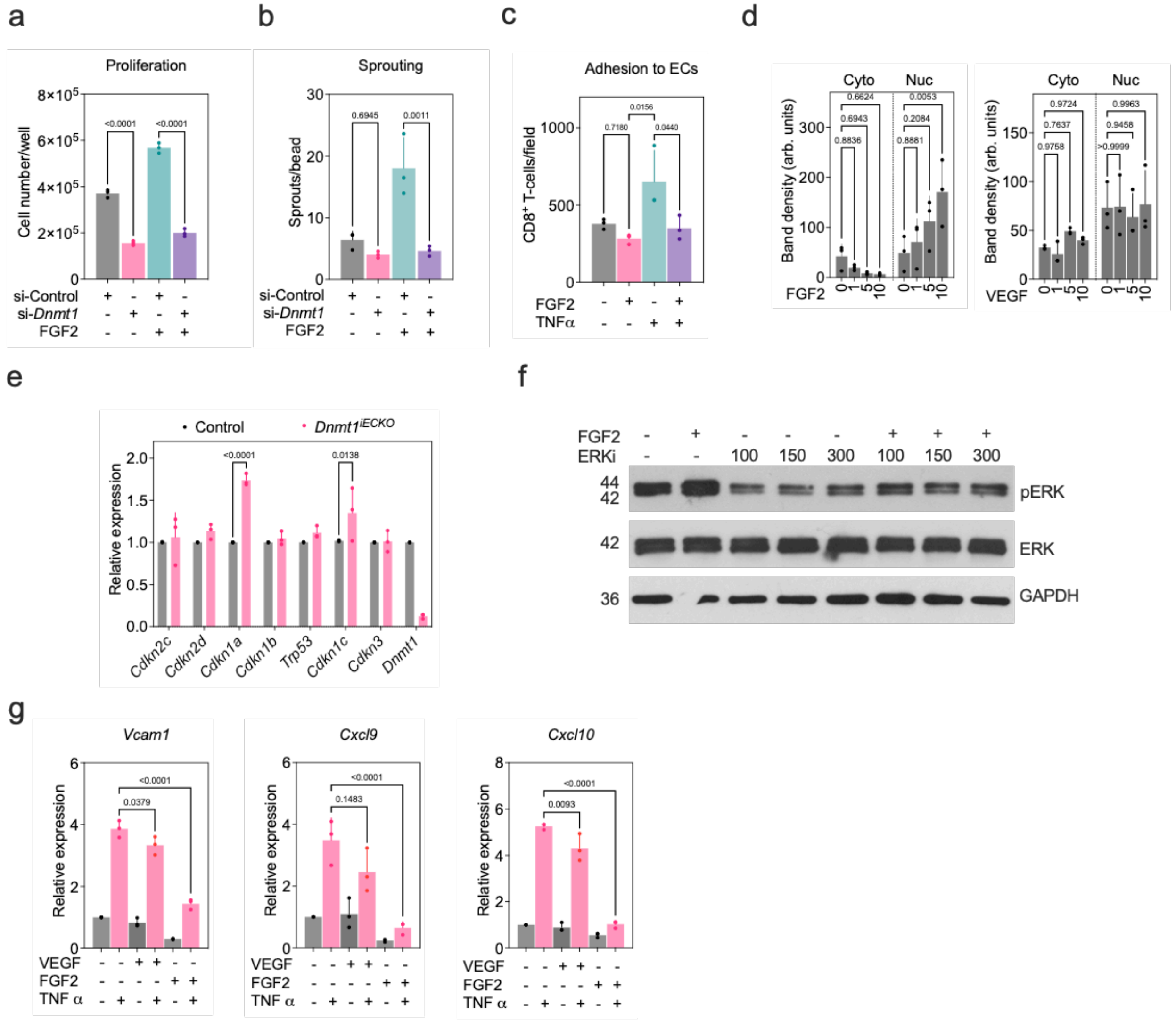

Supplementary Fig. 18.
(a) Cell growth assay using MGECs with the indicated treatment strategy (*n=3* independent experiments for quantification). Results were analyzed using ANOVA followed by Tukey's multiple comparisons test. (b) In vitro angiogenesis sprouting assay using MGECs with the indicated treatments. Each data point represents an individual EC-coated agarose bead (*n=3* biological replicates). Results were analyzed using ANOVA followed by Tukey's multiple comparisons test. (c) CD8 T-cell adhesion assay using MGEC monolayers under the indicated condition. Each data point is the mean of *n=3* biological replicates. Results were analyzed using ANOVA followed by Tukey's multiple comparisons test. (d) Quantification of band densities using three independent western blots under the indicated conditions (FGF2 versus VEGF treated). Results were analyzed using ANOVA followed by Tukey's multiple comparisons test. (e) qPCR analysis of cell cycle marker genes in MGECs in control versus *Dnmt1*-siRNA cultures. Each data point is the mean of *n=3* biological replicates run in triplicate. Results were

analyzed using ANOVA followed by Sidak’s multiple comparisons test. (f) Confirmatory western blot showing diminished pERK expression using the ERK inhibitor (representative of *n=3* independent blots). (g) qPCR analysis in MGECs under the indicated condition. Each data point is the mean of *n=3* biological replicates assayed in triplicate. Results were analyzed using ANOVA followed by Tukey’s multiple comparisons test. All error bars are mean -/+ STD. Source data are provided as a Source Data file.
