# Supplementary material for: Priming a vascular-selective cytokine response permits CD8+ T-cell entry into tumors
Source: Nat Commun. 2023 Apr 14;14:2122. doi: 10.1038/s41467-023-37807-z (PMC10101959; doi:10.1038/s41467-023-37807-z)
Supplement: Supplementary file 2 — Description of Additional Supplementary Files [file 41467_2023_37807_MOESM2_ESM.pdf]

Description of Additional Supplementary Files

Supplementary Data 1: Primer sequences used in this study.
