# Supplementary material for: Priming a vascular-selective cytokine response permits CD8+ T-cell entry into tumors
Source: Nat Commun. 2023 Apr 14;14:2122. doi: 10.1038/s41467-023-37807-z (PMC10101959; doi:10.1038/s41467-023-37807-z)
Supplement: Supplementary file 4 — Reporting Summary [file 41467_2023_37807_MOESM4_ESM.pdf]

nature portfolio

Corresponding author(s): Dudley, Andrew C

Last updated by author(s): Apr 27, 2023

Reporting Summary

Nature Portfolio wishes to improve the reproducibility of the work that we publish. This form provides structure for consistency and transparency in reporting. For further information on Nature Portfolio policies, see our Editorial Policies and the Editorial Policy Checklist.

Statistics

For all statistical analyses, confirm that the following items are present in the figure legend, table legend, main text, or Methods section.

n/a	Confirmed	
[ ]	[x]	The exact sample size (*n*) for each experimental group/condition, given as a discrete number and unit of measurement
[ ]	[x]	A statement on whether measurements were taken from distinct samples or whether the same sample was measured repeatedly
[ ]	[x]	The statistical test(s) used AND whether they are one- or two-sided<br>*Only common tests should be described solely by name; describe more complex techniques in the Methods section.*
[x]	[ ]	A description of all covariates tested
[ ]	[x]	A description of any assumptions or corrections, such as tests of normality and adjustment for multiple comparisons
[ ]	[x]	A full description of the statistical parameters including central tendency (e.g. means) or other basic estimates (e.g. regression coefficient) AND variation (e.g. standard deviation) or associated estimates of uncertainty (e.g. confidence intervals)
[ ]	[x]	For null hypothesis testing, the test statistic (e.g. *F*, *t*, *r*) with confidence intervals, effect sizes, degrees of freedom and *P* value noted<br>*Give P values as exact values whenever suitable.*
[x]	[ ]	For Bayesian analysis, information on the choice of priors and Markov chain Monte Carlo settings
[x]	[ ]	For hierarchical and complex designs, identification of the appropriate level for tests and full reporting of outcomes
[x]	[ ]	Estimates of effect sizes (e.g. Cohen's *d*, Pearson's *r*), indicating how they were calculated

Our web collection on statistics for biologists contains articles on many of the points above.

Software and code

Policy information about availability of computer code

Data collection	Single Cell Portal (Broad Institute)
Data analysis	Seurat R package (v 4.0) and Loupe browser (1OX genomics v 2.0)

For manuscripts utilizing custom algorithms or software that are central to the research but not yet described in published literature, software must be made available to editors and reviewers. We strongly encourage code deposition in a community repository (e.g. GitHub). See the Nature Portfolio guidelines for submitting code & software for further information.

Data

Policy information about availability of data

All manuscripts must include a data availability statement. This statement should provide the following information, where applicable:

- Accession codes, unique identifiers, or web links for publicly available datasets
- A description of any restrictions on data availability
- For clinical datasets or third party data, please ensure that the statement adheres to our policy

The scRNAseq data generated in this study have been deposited in the NCBI GEO database at GSE186467. The publicly available scRNAseq data generated by Hua et al. (2022) have been deposited in the NCBI GEO database at GSE198080 41. The publicly available scRNAseq data generated by Wu et al. (2021) have been deposited at (https://zenodo.org/record/5031502#.Y_Og3i2cbUI). The publicly available scRNAseq data generated by Wu et al. (2020) have been deposited at the European Nucleotide Archive (ENA) PRJEB35405. The remaining data are available within the Article, Supplementary Information, or Source Data file.

Human research participants

Policy information about studies involving human research participants and Sex and Gender in Research.

Reporting on sex and gender	N/A
Population characteristics	N/A
Recruitment	N/A
Ethics oversight	N/A

Note that full information on the approval of the study protocol must also be provided in the manuscript.

Field-specific reporting

Please select the one below that is the best fit for your research. If you are not sure, read the appropriate sections before making your selection.

☒ Life sciences ☐ Behavioural & social sciences ☐ Ecological, evolutionary & environmental sciences

For a reference copy of the document with all sections, see nature.com/documents/nr-reporting-summary-flat.pdf

Life sciences study design

All studies must disclose on these points even when the disclosure is negative.

Sample size	qPCR samples are run in technical triplicates with three biological replicates. Mouse tumor experiments were performed using a minimum of 5 mice per group.
Data exclusions	No data were excluded.
Replication	Three biological replicates were performed or a sufficient number of mice were used based on a power calculation. All replication attempts were successful.
Randomization	Mice were randomized into the different treatment groups based on their genetic background (control versus KO mice) and then treated with different drug combinations (e.g. checkpoint blockade).
Blinding	Blinding was used when feasible for quantification of tumor volumes using calipers or immunohistochemistry using ImageJ.

Reporting for specific materials, systems and methods

We require information from authors about some types of materials, experimental systems and methods used in many studies. Here, indicate whether each material, system or method listed is relevant to your study. If you are not sure if a list item applies to your research, read the appropriate section before selecting a response.

Materials & experimental systems

n/a	Involved in the study
☐	☒ Antibodies
☐	☒ Eukaryotic cell lines
☒	☐ Palaeontology and archaeology
☐	☒ Animals and other organisms
☒	☐ Clinical data
☒	☐ Dual use research of concern

Methods

n/a	Involved in the study
☐	☐ ChIP-seq
☐	☒ Flow cytometry
☐	☐ MRI-based neuroimaging

Antibodies

Antibodies used	anti-Dnmtl cern (Cat no ab 87654, Dilution 1:1,000, Abeam), anti-Dnmt1 (Cat no ab13537, Clone 60b1220.1, Abcam), anti-5MC (Cat no<br>28692, Clone D3S2Z, Dilution 1:100, Cell Signaling), anti-GAPDH (Cat no 5174S, Dilution 1:2,000, Cell Signaling), anti-Histone H3 (Cat no<br>9715, Dilution 1:1000, Cell Signaling), anti-ERK (Cat no 9102s, Dilution 1:1,000, Cell Signaling),

anti-phospho-ERK (Cat no 4370s, Dilution 1:1,000, Clone D13.14.4E, Cell signaling), anti-SMA (Cat no A5228, Dilution 1:200, Clone A14,
Sigma), PE-anti-mouse CD8 (Cat no 100707, Dilution 1:50, BioLegend), InVivoPlus anti-mouse CD8 (Cat no BP0061, Clone 2.43, Dilution
20 mg/Kg, BioXcell), CD8a monoclonal antibody(Cat no14-0081-82, Clone 53-6.7, Dilution 1:50, eBiosciences), anti-CDllc (Cat no 550283, Clone HL3, Dilution 1:50, BD Biosciences), PE-anti-mouse CD11c (Cat no 561044, Clone HL3, Dilution 1:50, BD Pharmingen), APC
anti-CD45 (Cat no 559864, Clone 30-F11, Dilution 1:50, BD Pharmingen), anti-CD31 (Cat no 553373, Clone MEC13.3, Dilution 1:20, BD Pharmingen), anti-F480 (Cat no MCA497PET, Dilution 1:50, AbD serotec), anti-Meca79 (Cat no 553863, Dilution 1:50, BD Biosciences) ,
anti-Vcaml (Cat no 550547, Clone 429, MVCAM.A, Dilution 1:50, BD Biosciences), PE anti-Vcam1(Cat no 561613, Dilution 1:100, BD Pharmingen), PE-CD62E (Cat no 553751, Dilution 1:50, Clone 10e9.6, BD
Pharmingen), anti-CD4 (Cat no 561836, Clone RM4-5, BD Biosciences), anti-CD183 (Cat no 126517, Clone CXCR3-173, Dilution 1:50, BioLegend), anti-granzyme B (Cat no 17215, Clone D2H2F, Dilution 1:100, Cell Signaling), anti-mouse lgG (Cat no, BE0083, BioXcell), anti-PD-1 (Cat no BE0146, Clone CD279, BioXcell), anti-CTLA-4 (Cat no BP0131, CD152, BioXcell), and anti-PD-Ll
(Genentech, MTA program).

Validation: Antibodies are validated by the manufacturer.

Eukaryotic cell lines

Policy information about cell lines and Sex and Gender in Research

Cell line source(s): EO771 (CH3 Biosystems) , PyVMT (ATCC), HUVEC (Lonza cC2517AS), Human lung fibroblasts (Lonza CC-2512), JURKAT (TIB-152, modifications described in Kitajima et al., Cancer Discovery, 2019), MGECs, HEK293 (ATCC).

Authentication: E0771 cell line is authenticated by CH3 Biosystems using short tandem repeat profiling.

Mycoplasma contamination: Cell lines were routinely tested for mycoplasma and cultured with a maintenance dose of plasmocin.

Commonly misidentified lines (See ICLAC register): N/A

Animals and other research organisms

Policy information about studies involving animals; ARRIVE guidelines recommended for reporting animal research, and Sex and Gender in Research

Laboratory animals: C57BL6 female mice (Postn-CreERT2, VeCadh-cre;ZsGreen, and VeCadh-Cre;DNMT1iECKO) at 8 wks. of age. Light cycle : A 14 hr light/10 hr dark cycle. Temp and humidity: 65-75 F with 40-60% humidity. Standard lab chow fat content ranges from 4% to 11%. Water: accessible at all times.

Wild animals: N/A

Reporting on sex: Females were used because mammary tumor lines were tested in vivo.

Field-collected samples: N/A

Ethics oversight: N/A

Note that full information on the approval of the study protocol must also be provided in the manuscript.

ChIP-seq

Data deposition

☐ Confirm that both raw and final processed data have been deposited in a public database such as GEO.

☐ Confirm that you have deposited or provided access to graph files (e.g. BED files) for the called peaks.

Data access links
May remain private before publication.: N/A

Files in database submission: N/A

Genome browser session (e.g. UCSC): N/A

Methodology

Replicates: N/A

Sequencing depth	N/A
Antibodies	N/A
Peak calling parameters	N/A
Data quality	N/A
Software	N/A

Flow Cytometry

Plots

Confirm that:

- [x] The axis labels state the marker and fluorochrome used (e.g. CD4-FITC).
- [x] The axis scales are clearly visible. Include numbers along axes only for bottom left plot of group (a 'group' is an analysis of identical markers).
- [x] All plots are contour plots with outliers or pseudocolor plots.
- [x] A numerical value for number of cells or percentage (with statistics) is provided.

Methodology

Sample preparation	Tissues homogenized in collagenase/dispase/DNAse for 60 mins. at 37C. Samples further disrupted using the Miltenyi tissue homogenizer. Final samples filtered through a 100 uM strainer, washed, and resuspended in FACS buffer before antibody staining.
Instrument	Accuri C6 and FACS Caliber (BD)
Software	FloJo V 10.6
Cell population abundance	Purity based on fluorescence markers used such as APC and PE which are well separated and well resolved. Any doublets identified were aborted.
Gating strategy	Gates established using FMO controls (sample that does not exhibit ZSGreen). FSC and side scatter plots were used to remove debris. FCS and trigger Pulse width was used to identify singlets. qPCR for lineage-based markers were used postsorting<br>to validate purity. A sort mode of 1.0 drop pore (high purity) was used for all sorting.

- [x] Tick this box to confirm that a figure exemplifying the gating strategy is provided in the Supplementary Information.

Magnetic resonance imaging

Experimental design

Design type	N/A
Design specifications	N/A
Behavioral performance measures	N/A

Acquisition

Imaging type(s)	N/A
Field strength	N/A
Sequence & imaging parameters	N/A
Area of acquisition	N/A

Diffusion MRI ☐ Used ☒ Not used

Preprocessing

Preprocessing software	N/A
Normalization	N/A

Normalization template	N/A
Noise and artifact removal	N/A
Volume censoring	N/A

Statistical modeling & inference

Model type and settings	N/A
Effect(s) tested	N/A

Specify type of analysis: ☐ Whole brain ☐ ROI-based ☐ Both

Statistic type for inference (See Eklund et al. 2016)	N/A
Correction	N/A

Models & analysis

n/a	Involved in the study
☒	☐ Functional and/or effective connectivity
☒	☐ Graph analysis
☒	☐ Multivariate modeling or predictive analysis
